# Patient-reported outcome measures in children, adolescents, and young adults with palliative care needs—a scoping review

**DOI:** 10.1186/s12904-023-01271-9

**Published:** 2023-10-06

**Authors:** Heidi Holmen, Anette Winger, Simen A. Steindal, Kirsti Riiser, Charlotte Castor, Lisbeth Gravdal Kvarme, Kari L. Mariussen, Anja Lee

**Affiliations:** 1 Department of Nursing and Health Promotion, Oslo Metropolitan University, St. Olavs Place, Post Box 4, 0130 Oslo, Norway; 2 Lovisenberg Diaconal University College, Lovisenberggt, 15B, 0456 Oslo, Norway; 3 Faculty of Health Studies, VID Specialized University, Oslo, Norway; 4 Department of Rehabilitation Science and Health Technology, Oslo Metropolitan University, St. Olavs Place, Post Box 4, 0130 Oslo, Norway; 5 Department of Health Sciences, Lund University, Box 157, 221 00 Lund, Sweden; 6 Division of Pediatric and Adolescent Medicine, Oslo University Hospital HF, Nydalen, Box 4950, 0424 Oslo, Norway

**Keywords:** Patient-reported outcome measures, Pediatric palliative care, Health and psychosocial instruments, Scoping review, Symptom assessment

## Abstract

**Background:**

Measuring outcomes facilitates evaluation of palliative services for children, adolescents, and young adults (CAYAs) with life-limiting and/or life-threatening (LL/LT) conditions. Implementation of patient-reported, proxy-reported, or patient-centered outcome measures (hereafter PROMs) is recommended to ensure palliative services. The purpose of this scoping review was to provide an overview of PROMs relevant for CAYAs living with LL/LT conditions eligible for pediatric palliative care (PPC).

**Methods:**

Arksey and O’Malley’s 6-stage scoping review framework was used to guide the review. The identified citations had to report on PROMs in any context including CAYAs with LL/LT conditions up to 25 years of age. A systematic search of Medline, EMBASE, CINAHL, APA PsycInfo, Health and Psychosocial Instruments, and AMED took place in January 2021 and was updated in June 2022. Citations were screened independently by pairs of researchers. The scoping review protocol was registered, and peer-review published.

**Results:**

Of 3690 identified citations, 98 reports were included, of which the majority were from Western countries and about PROMs in CAYAs living with cancer or organ failure. A total of 80 PROMs were identified, assessing a range of phenomena, where quality of life and symptoms (especially pain) during the stage of ongoing care were the most frequent. There were only a few reports about outcome measures at time of diagnosis or in end-of-life care. CAYAs self-reported on the PROMs or collaborated with their parents in about half of the reports, while the remaining had proxies answering on behalf of the CAYAs. In the identified reports, PROMs were used to characterize a sample through cross-sectional or longitudinal research, and less often to assess effects of interventions.

**Conclusion:**

The identified PROMs in the CAYA population eligible for PPC is characterized by studies in high-income countries during ongoing care, primarily in patients with cancer or organ failure. More research is needed in patients living with other LL/LT conditions, and during different stages of the disease course, especially at time of diagnosis, during transition to adulthood, and in end-of-life care. This scoping review of PROMs relevant for young patients eligible for PPC may inform future research about patient-/proxy-reported or patient-centered outcome measures in PPC.

**Trial registration:**

Review registration: (https://osf.io/yfch2/) and published protocol (Holmen et al. Syst Rev. 10:237, 2021).

**Supplementary Information:**

The online version contains supplementary material available at 10.1186/s12904-023-01271-9.

## Background

Pediatric palliative care (PPC) is a holistic concept defined as the prevention and relief of suffering in children, adolescents and their families when facing a life-limiting and/or life-threatening (LL/LT) condition [1–3]. The ultimate goal of PPC is to improve the quality of life (QOL) and promote dignity and comfort for the child and the child’s family at all levels of the health care services [2]. The World Health Organization (WHO) states that the integration of PPC into public healthcare systems is essential to achieve the Sustainable Development Goal on universal health coverage [3].

To strengthen the evidence of the benefits of integrated PPC services, the effectiveness of PPC must be addressed by demonstrating outcomes [4, 5], as an *“outcome denotes the effects of care on the health status of patients and populations*” [6]. A “patient-reported outcome” represents any report from the patient regarding a health phenomenon, such as QOL, pain or other symptoms [7]. Patient-reported outcome measures (PROMs) are the measures or methods used to collect these reports. Whenever possible, self-reporting is the best way to assess the effect of treatment and care [3, 8]. However, in PPC, major groups of patients (infants, children, and adolescents with cognitive impairment or in critical stages of illness) may be unable to self-report. In such circumstances, proxy reports conducted by family members or healthcare personnel (HCP) may be helpful [9]. Such proxy reports may be referred to as “family-centered outcome measures” [10] or “patient-centered outcome measures [11]. In this review, the term PROM will be used to describe outcome measures focusing on the well-being of the patient, whether they are reported directly by the patient, or indirectly by family members or HCP.

Core outcomes in PPC have been identified by several researchers [12–14]. In 2018, Downing et al. undertook a narrative review of outcome measurement in PPC, focusing on the development of a multi-dimensional outcome scale for PPC — the African C-POS [14]. Friedel et al. [15] did a systematic review of instruments used to assess the impact of PPC interventions, however, only 19 of 2150 articles met the eligibility criteria, and only five of 23 reported instruments included patient-reported (child) outcome measures. Some reports within the field of outcome research have focused on specific diagnostic groups, especially childhood cancer [16] or specific phenomena, such as health-related quality of life (HRQL) [17] and quality of end-of-life care [18]. A thorough realist review has studied the contexts and mechanisms through which beneficial outcomes, from the perspectives of parents, can be achieved [19]. Childhood cancer has been the dominating diagnostic group within PROMs research in PPC [16, 20–22], while in other groups, such as pediatric cardiology, the very lack of outcome or measurement tools has been identified as the top barrier for symptom relief [23].

The UK charity Together for Short Lives (TfSL) has defined care pathways for children, adolescents, and young adults (CAYAs) living with LL/LT conditions. These pathways provide frameworks for specific stages of illness, from perinatal care and time of diagnosis or recognition of a LL/LT condition, through the stage of ongoing care and transition to end-of-life care [24]. Young patients and their families need tailormade support throughout the entire disease course, and care providers need tools for systematic feedback from those in need of treatment, care, psychosocial and existential support. The use of PROMs could provide this kind of feedback.

A scoping study is a type of literature review that differs from systematic reviews by addressing relatively broad topics, while not asking very specific research questions [25]. Scoping reviews are considered to be of particular relevance to disciplines where randomized controlled trials are mostly lacking, making it difficult to undertake systematic review [26], as is the case in the emerging field of PROMs research in PPC*.* The majority of young patients living with LL/LT conditions are not receiving PPC [3], while outcome measurements applicable for the large and diverse population of CAYAs living with LL/LT conditions (including proxy reporting) is expanding rapidly. Thus, a scoping review of research on PROMs relevant for CAYAs living with diagnoses previously included in the definition of LL/LT conditions [27] is warranted.

The aim of this review was to provide an overview of PROMs which may be applicable for the large and diverse population of CAYAs living with LL/LT conditions eligible for PPC.

## Methods

### Design

The framework by Arksey and O’Malley [25] was applied by following six steps: 1) identifying the research question; 2) identifying relevant studies; 3) study selection; 4) charting the data; 5) collating, summarizing and reporting the results; and 6) consultation exercise, along with the later methodological improvements suggested by Levac et al. [26]. The scoping review report followed the Preferred Reporting Items of Systematic Reviews extension for Scoping reviews (PRISMA ScR) checklist [28]. Deviations from the published protocol [29] and registered protocol (https://osf.io/yfch2/) have been described in Supplementary File 2.

#### Step 1: Identifying the research question

In contrast to a systematic review, a scoping review should have a broad research question [25, 28, 30]. The research question of this review was a result of discussions among researchers, clinicians, stakeholders, and user representatives associated with the Norwegian research network Children in Palliative Care (CHIP). Based on clinical experience, previous research by network members [31, 32] and international research priorities in PPC [33], the group found that a scoping review of PROMs in PPC could contribute 1) to obtain an overview of tools used to monitor clinical outcomes in CAYAs with LL/LT conditions, and 2) to identify knowledge gaps relevant for future research.

The research questions were as follows: What is known from the published, peer-reviewed reports (studies) about PROMs for CAYAs eligible for PPC? Which approaches exist for systematic outcome measurement in CAYAs eligible for PPC, including tools for proxy report when patients are unable to self-report?

#### Step 2: Identifying relevant studies

##### Eligibility criteria

The population, concept, and context tool [30] guided our eligibility criteria (Table 1). We included reports on CAYAs with diagnoses indicating a need for PPC, regardless of whether the patients were actually included in a formal PPC-program or not. Primary research in peer-reviewed reports published in scientific journals, as well as reports on development, use, or evaluation of PROMs in PPC were included, both from research and clinical settings. All kinds of tools and modes of assessment were included, regardless of study design. Due to the inability of some CAYAs to self-report, studies based on proxy-reporting (a.k.a. family-centered or patient-centered outcome measures) were also included. Young adults up to 25 years of age were included because several studies in PPC have applied this age range [34–36].

**Table 1:** Population, concept, and context [29]

	Scoping review target
Population	Children, adolescents, and young adults (CAYAs) aged 0–25 years eligible for PPC
Concepts	PROMs to assess symptoms, care needs and/or burden, reported either by the patient, caregivers, or HCP (proxy reports)
Context	The patient may be cared for at any level of the healthcare services, at home, or included in a research setting

*CAYAs* Children, adolescents, and young adults, *HCP* healthcare personnel, *PPC* pediatric palliative care, *PROMs *patient reported outcome measures

Reports solely on PROMs for adults were excluded, while papers including both children and adults were included if PROMs relevant to CAYAs could be extracted from the report. In order to describe the entire range of published reports relevant for our research questions, there were no limitations on dates of publication. Papers in English, German, or Scandinavian were included, while other languages were excluded because of a lack of resources for translations. Grey literature was not included in this scoping review as we aimed to describe the entire range of published reports (studies).Neither did we conduct reference list searches among the included reports.

#### Information sources and search

The first systematic search in the Medical Literature Analysis and Retrieval System Online (Medline), Excerpta Medica database (EMBASE), Cumulative Index to Nursing and Allied Health Literature (CINAHL), American Psychological Association (APA) PsycInfo, and Allied and Complementary Medicine Database (AMED) was conducted on January 26th, 2021, and in Health and Psychosocial Instruments (HaPI) on April 7th, 2021. The search was developed and tailored in collaboration with a librarian who had expertise in systematic searches in medical research databases and peer-reviewed research; the search was carried out according to the Peer Review of Electronic Search Strategies (PRESS) checklist [37]. The search was updated June 16th, 2022, to identify any new and eligible publications. All search strings can be found in Supplementary File 3.

#### Step 3: Data selection

When the final search was conducted, the search results were deduplicated using Endnote, and the remaining citations were imported to the screening and extraction tool Covidence [38]. The screening criteria’s clarity was piloted by assessing the citations after the duplicates were removed. In this pilot study, 10% of the sample was screened by title and abstract by two independent researchers (HH and SAS), which was equivalent to 300 citations each. This resulted in eight conflicts, which led to a discussion and rewording of the eligibility criteria for clarity. Then, all citations were randomly screened based on title and abstract by two independent reviewers, each using the automatic assignment functions of Covidence, allowing random assignment of citations to ensure dual assessment of all citations by two independent reviewers. The full-text screening followed the same independent, blinded, and random assignments of citations through Covidence [38]. Two researchers (HH and AL) resolved the conflicts consecutively.

#### Step 4: Charting the data

##### Data extraction

The research team developed a specific extraction template to gather the necessary data to answer the study aim (Table 2), and the template was implemented in Covidence extraction 2.0 [38].

**Table 2:** Data extraction template

General information	Methods	Participants	Respondents to PROM	PROM	Results
Author and year, country	Primary aim of the study Whether the aim was to: 1) Develop a new PROM 2) Test a PROM 3) Evaluate an intervention with PROM 4) Assess the characteristics of a population with PROM	Population, sample size and age Group according to Together for Short Lives (TfSL).^a^ Care pathway according to TfSL^b^	Self-report, proxy, both self-report and proxy, other, specify in text	Target group and age of original PROM Whether the PROM was: 1) Developed for adults and tailored to children 2) Developed for children 3) Developed as a proxy measure 4) Unknown/ not clear Experiences with PROM (sensitive/ responsive/ any comments/ limitations)	Results of interest to our aim

*PROM* Patient reported outcome measures, *TfSL* Together for Short Lives

^a^Patient groups as defined by TfSL [39]; Group 1: Life-threatening conditions for which curative treatment may be feasible but can fail; Group 2: Conditions in which premature death is inevitable. Treatment may aim to prolong life and allow normal activities; Group 3: Progressive conditions without curative treatment options. Treatment exclusively palliative may extend over many years; and Group 4: Irreversible but nonprogressive conditions causing severe disabilities leading to susceptibility to health complications and likelihood of premature death

^b^Care pathways defined by TfSL [24]; Care pathway 1: Time of diagnosis or recognition of LL/LT condition; Care pathway 2: During ongoing care; Care pathway 3: End of life care; Care pathway 0: Perinatal care; and Care pathway 4: During transition to adulthood

Following the extraction template, pairs of researchers extracted the data, where one researcher extracted data and the second controlled the extracted data and approved or changed the extraction. When completed, all data were downloaded by HH from Covidence in an Excel file for further work. HH and AL assured that all populations and PROMs were according to our inclusion criteria before the final data file was analyzed.

#### Step 5: Collating, summarizing, and reporting the results

According to the scoping review protocol, our intention was to summarize the data using the first two steps of thematic synthesis [40]. However, because of the nature and number of findings, the data was organized according to the data extraction table; this was done before summarizing and controlling the identified PROMs with their details. In the cases where data from one study were reported in separate reports, the data were extracted per report. In order to present the diagnoses in the reports, we grouped the diagnoses according to the four categories for LL/LT conditions, as defined by Together for Short Lives (TfSL) [39]: 1) life-threatening conditions for which curative treatment may be feasible but can fail, 2) conditions where premature death is inevitable, 3) progressive conditions without curative treatment options, and 4) irreversible but nonprogressive conditions causing severe disabilities leading to susceptibility to health complications and likelihood of premature death.

##### Critical appraisal of individual sources of evidence

In line with Arksey and O’Malley [25] and the PRISMA ScR checklist [28], methodological appraisal of the included studies has not been conducted because the aim was to scope the available evidence, not systematically assess its methodological quality.

#### Step 6: Consultation exercise

The preliminary results of the scoping review were presented to stakeholders within palliative care for children as part of the consultation exercise described by Arksey and O’Malley [25]. We aimed at presenting preliminary findings to stakeholders with an interest in PPC and those interested in PROMs, or both. In these meetings, a brief presentation of scope and findings were presented by either the first or last author, followed by an open discussion. Notes were taken, and the professional background and number of participants was collected.

## Results

### Study selection

**Fig. 1:**
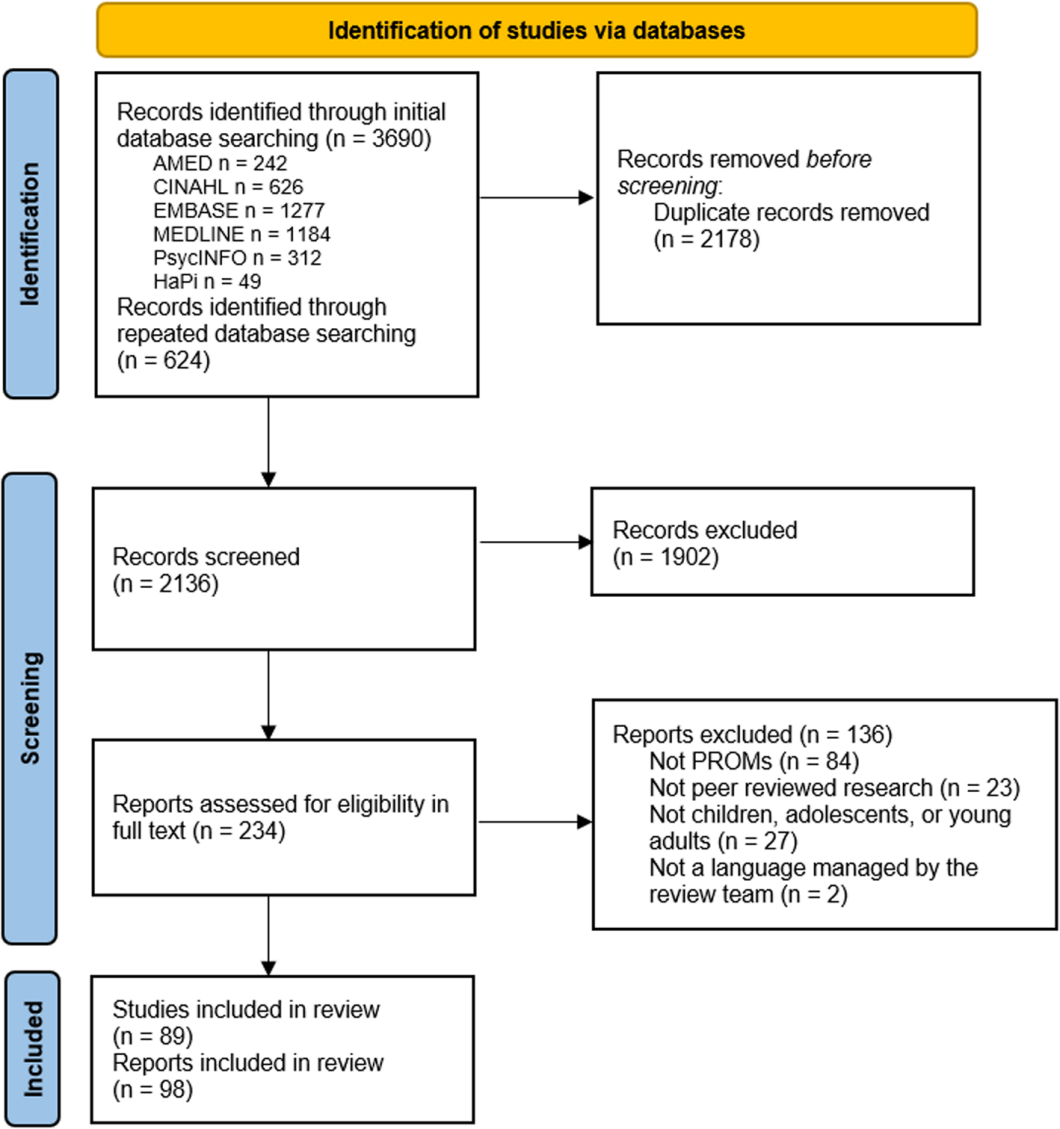
PRISMA flow diagram of the search and screening process

Initially, 1807 unique reports were identified, and another 329 reports were identified in the repeated search, adding up to a total of 2136 reports to screen. After screening, 234 reports were read in full text, resulting in 98 reports presenting results of 89 studies published between 2004 and 2021 being included in the scoping review analysis [35, 36, 41–136] (Fig. 1. Flow diagram). Supplementary details of the included reports can be found in Supplemental File 4: Extracted Data.

### Characteristics of the identified PROMs

A total of 80 PROMs were found in the included studies (Table 3). For more information on each PROM and full reference we refer to Supplementary File 5.

**Table 3:** Phenomena covered by the identified PROMs (*n* = 80)

Phenomena	N of PROMs	PROM name
Adverse events/ side effects	2	Pediatric Patient-Reported Outcomes version of the Common Terminology Criteria for Adverse Events (Ped-PRO-CTCAE); study specific [79]
Anger (co-occurring symptoms)	1	Patient-Reported Outcomes Measurement Information System (PROMIS) pediatric Anger
Anxiety symptoms	3	McMurtry Faces Anxiety Scale; Patient-Reported Outcomes Measurement Information System (PROMIS) Anxiety; Screen for Anxiety Related Emotional Disorders (SCARED)
Anxiety and depressive symptoms	2	Hospital Anxiety and Depression Scale (HADS); Patient-Reported Outcomes Measurement Information System (PROMIS) depressive symptoms
Anxiety and pain intensity	1	Children’s Anxiety and Pain Scale (CAPS)
Benefit (and burden)	2	Benefit and Burden Scale for Children; Benefit Finding Scale for Children (BFSC)
Comfort	3	Children’s comfort Daisies; Children’s Comfort Line Visual Analog Scale; Children’s Comfort 1-item
Depressive symptoms	1	Children’s Depression Inventory (CDI)
Distress	2	Distress Thermometer (DT); Kessler 6 Psychological Distress Scale (K-6)
Dyspnea	1	Dalhousie Dyspnea Scale
Fatigue	2	Childhood Fatigue Scale; Patient-Reported Outcomes Measurement Information System (PROMIS) fatigue
Health status	2	Child health status; study specific [100]
Health-related quality of life (HRQL) generic	4	Children’s Health Questionnaire; Kidscreen-52; PedsQL core; TNO-AZL questionnaire for children’s health-related quality of life (HRQL)
HRQL disease specific	6	PedsQL Brain Tumor Scale; PedsQL Cancer Module; PedsQL End-Stage Renal Disease (ESRD); PedsQL Fatigue Scale; PedsQL Neuromuscular Module; Quality of Life for Primary Ciliary Dyskinesia (QOL-PCD)
HRQL and symptoms	1	PediQuest (PQ) with PedsQL and Memorial Symptom Assessment Scale (MSAS)
Hope	1	Snyder Hope Scale
Life threat	1	Study specific [104]
Mobility	1	Patient-Reported Outcomes Measurement Information System (PROMIS) mobility
Multidimensional	1	Patient-Reported Outcomes Measurement Information System (PROMIS) profile
Nausea	1	Baxter Retching Faces (BARF)
Pain	6	Brief Pain Inventory (BPI); Childhood Health Assessment Questionnaire Pain (CHAQ-pain); Face, Legs, Activity, Cry, Consolability Healthcare Personnel (FLACC HCP); Faces pain scale; Patient-Reported Outcomes Measurement Information System (PROMIS) Health Assessment Pain; Wong-Baker Faces
Pain, agitation, and sedation	1	Nurse-Documented Neonatal Pain, Agitation and Sedation Scale (N-PASS)
Pain characteristics and management	1	Study specific [48]
Pain and distress	1	COMFORT-B scale
Pain intensity	1	Patient-Reported Outcomes Measurement Information System (PROMIS) pain intensity
Pain interference	1	Patient-Reported Outcomes Measurement Information System (PROMIS) pain interference
Pain location	1	Body outline
Palliative outcomes	1	Children’s Palliative Outcome Scale (CPOS)
Posttraumatic stress disorder reaction	1	Posttraumatic Stress Disorder Reaction Index (PTSDI)
Psychological stress	1	PROMIS Pediatric Psychological Stress
QOL	7	Generic Children's QOL Measure (GCQ); General Health Assessment for Children (GHAC); Inventory for the Assessment of the QOL in children and adolescents (ILK); Pediatric Advanced Care-Quality of Life Scale (PAC-QoL); Scheduled evaluation of individual quality of life (SEIQoL); Short-Form-36 (SF-36); Vécu et Santé Perçue de l’Adolescent et l’Enfant (VSP-A)
QOL disease specific	3	Preschool Pediatric Cardiac Quality of Life Inventory; Sinus and Nasal Quality of Life Survey (SN-5); COPD assessment test (CAT)
Resilience	1	Connor Davidson Resilience Scale (CDRISC-10)
Suffering	1	Study specific [51]
Symptoms (physical and psychological)	7	ePROtect; Memorial Symptom Assessment Scale (MSAS); PediQuest Memorial Symptom Assessment Scale (PQ-MSAS); study specific [84, 117, 124]; Symptom Screening in Pediatrics Tool (SSpedi)
Symptoms (psychological)	1	Brief symptom inventory
Symptoms and suffering	3	Study specific for infants [45]; study specific [80]; study specific [106]
Symptoms at end of life	1	Study specific [97]
Symptoms related to therapy	1	Therapy-Related Symptom Checklist (TRSC);
Well-being	2	Nurse Perceptions of Infant Well-Being Survey; “To lose a child”;

Patient reported outcome measures (PROMs). Only study-specific PROMs developed within the included studies are cited in this table because citing the standardized PROMs would require searching and citing beyond the scope of the included papers. For a complete overview of which PROMs that has been applied in which studies, we refer to the supplementary file 5

The total number of PRO items were rarely summarized in the reports. In the reports (*n* = 47) providing items per PROM, there were a range from 3 to 390 items (median 34 items) [35, 41, 42, 44, 50–52, 54, 56, 57, 62, 63, 66, 68, 71, 73, 80–82, 85–87, 90–92, 94, 95, 97, 98, 100, 102, 103, 107, 110, 112, 114–119, 124, 126–128, 134, 136]. In 30 reports, the number of items was missing for one or more of the identified PROMs, and a total number was not possible to calculate [36, 43, 45, 48, 49, 55, 58–61, 65, 67, 70, 75–77, 83, 93, 99, 104, 106, 108, 109, 120, 122, 123, 125, 130, 132, 133]. In ten studies, the number of items varied by age group or who chose to respond [46, 72, 79, 84, 88, 89, 96, 105, 111, 122]. One or more PROMs used in nine studies were responsive, and new questions were presented to the respondent based on the previous answers; thus, the total number of items could be found [47, 53, 64, 69, 74, 78, 101, 131, 135]. One report [129] used visual PROMs only, which were delivered as validated worksheets for the individual symptoms reported.

The PROMs were administered in several ways, such as through paper-, interview-, tablet-, or computer-based methods at home or in the clinic through a webpage or by e-mail. Furthermore, the expected time needed to answer was mentioned in 13 reports, ranging from 45 s to 2 h [54, 59–61, 67, 78, 84, 87, 91, 97, 105, 116, 133].

The most frequently described PROMs were various versions of the PedsQL (*n* = 47 reports/*n* = 44 studies), both the generic and disease-specific versions or a combination of them [36, 41–44, 50, 52, 56, 62, 63, 67, 72, 74, 77, 85, 91–95, 98–104, 107, 110–112, 114, 115, 123, 126–128, 135]. Twelve reports [45, 48, 51, 79, 80, 84, 97, 100, 104, 106, 117, 124] presented PROMs specifically developed to suit their specific study context.

PROMs were self-reported in 26 studies [35, 48, 49, 53, 55, 64, 65, 67, 69, 70, 73, 75–77, 86, 88, 105, 107, 109, 120, 121, 129, 131–134], by children–parent dyads in 32 studies [36, 41, 42, 44, 46, 50, 54, 56, 61, 63, 66, 71, 72, 78, 80, 81, 84, 90, 92–95, 103, 104, 110–112, 116, 118, 125, 127, 128, 136], and by proxy (parent or HCP) in nineteen studies [45, 47, 51, 58–60, 68, 79, 83, 85, 97, 98, 102, 106, 114, 115, 117, 124, 126]. In one study, both children, parents, and nurses answered the PROMs [130]. In the remaining studies, the respondents varied depending on the setting and the child’s condition [43, 57, 62, 74, 87, 89, 91, 96, 100, 101, 108, 123, 135].

### Characteristics of the participants in the included reports

The sample size of the included reports varied from six to 2500, with a total of 15,305 CAYAs. The median number of included CAYAs was *n* = 75. Among the 57 papers providing details of a parent sample [12, 36, 41, 42, 44–47, 50–54, 58, 60–63, 72, 74, 76, 78–80, 82, 84–86, 89, 93–100, 102, 103, 106, 108, 110–112, 114–118, 124, 125, 127, 128, 130, 133, 134], a total of 8,659 parents were included, with a median of 68 (min–max 10–1138) parents included in these samples. In 15 reports, HCPs were involved in answering the PROMs [59–61, 70, 90, 106, 111, 113, 121, 122, 124, 130, 132, 135, 136], but only five of these gave the number of HCPs (total of *n* = 404) included [59, 61, 90, 113, 132].

The inclusion criteria for age were reported in 86 papers, ranging from infants born at gestational age 23 weeks to patients 39 years old [35, 36, 41–44, 46–49, 52–59, 61–78, 80–82, 84–105, 107–113, 115, 116, 118–123, 125–130, 133–136]. Mean age was reported in 59 papers [35, 41, 44, 46–50, 52–57, 62–69, 71–73, 75, 76, 78, 80, 81, 83, 84, 88, 89, 91–96, 98, 103, 104, 107–109, 111, 119, 120, 123, 125–132, 134], ranging from 2 to 24 years, and the overall (not weighted) mean age was 12 years. The specific age of the included CAYAs was missing in 28 reports [36, 42, 43, 61, 70, 77, 79, 85, 86, 90, 100, 102, 105, 110, 112–116, 118, 121, 122, 133, 135, 136], but a pediatric, adolescent, or young adults’ sample was given. In the remaining reports, the CAYAs were either infants [45, 59, 60], previously deceased [51, 82, 97, 106, 117, 124], or their median age was provided [58, 87, 99].

When grouping the included reports according to TfSL categories [39], most studies were in TfSL category 1 with a range of phenomena measured by the identified PROMs (Table 4).

**Table 4:** TfSL category, diagnosis groups, and phenomena reported

TfSL categories	Conditions	N of studies	Phenomena reported in the identified PROMs
1) Life-threatening conditions for which curative treatment may be feasible but can fail	Cancer; Organ failure	70	Adverse events; benefit; combination of multiple phenomena; comfort; depression, fatigue; distress; general health; HRQL; QOL; pain; psychological stress; PTSD; resilience; sickness; and various symptoms
2) Conditions for which premature death is inevitable. Treatment may aim at prolonging life and allowing normal activities	Primary ciliary dyskinesia; HIV/AIDS	2	PCD-HRQL; HRQL; nasal-related HRQL; disease-specific QOL; QOL
3) Progressive conditions without curative treatment options. Treatment exclusively palliative and may extend over many years	Neurologic progressive disease	3	Fatigue; HRQL; neuromuscular HRQL; pain
4) Irreversible but nonprogressive conditions causing severe disabilities leading to susceptibility to health complications and likelihood of premature death		0	
A combination of categories	Various	23	Anxiety; distress; health status; HRQL; nausea; pain; palliative outcomes; QOL; suffering symptoms

*HRQL* Health-related quality of life, *PCD-HRQL* Primary ciliary dyskinesia health-related quality of life, *PROM* Patient-reported outcome measure, *PTSD* Post-traumatic stress disorder, *QOL* Quality of life, *TfSL* Together for short Lives

CAYAs with cancer were included in 48 reports [35, 36, 41, 43, 48, 53, 57, 64, 69, 70, 73–80, 84, 85, 87–89, 95–99, 101–104, 108, 109, 113–118, 121, 122, 125, 130, 131, 134–136], and patients with various types of organ failure such as kidney, liver, or heart failure were included in 22 reports [42, 44, 49, 50, 52, 54–56, 62, 63, 67, 68, 91–94, 105, 107, 110, 112, 119, 120]. Three reports included patients with progressive neurological conditions [65, 71, 128], while 23 reports included patients with various LL/LT conditions in combination [45, 47, 51, 58–61, 66, 72, 81, 82, 86, 90, 100, 106, 111, 123, 124, 126, 127, 129, 132, 133].

In terms of PPC care pathways [39] there were only two reports [59, 60] related to perinatal care, and three reports [53, 85, 95] regarding pathway 1 (at diagnosis or recognition of a LL/LT condition), 82 reports regarding pathway 2 (ongoing care) [35, 36, 41–44, 46–50, 52, 54–58, 61–65, 67–81, 83, 84, 86–94, 96, 98–105, 107–116, 119–123, 125, 127–129, 131–137] and nine reports related to pathway 3 (recognition of death approaching, end-of-life care, and bereavement support) [45, 51, 82, 97, 106, 117, 118, 124, 130]. Two reports presented findings including CAYAs from both pathway 2 and 3 [66, 126].

### Study design and primary aim of the included reports in terms of their use of PROM

Most (*n* = 30) of the included reports were cross-sectional studies [42–44, 47, 48, 51, 54, 56, 58, 63, 64, 67, 74, 81, 83, 86, 92, 94, 98, 104, 105, 107, 108, 112, 114, 115, 119, 120, 123, 127] or retrospective cross-sectional studies (*n* = 5) [82, 97, 106, 117, 124], followed by prospective cohorts or longitudinal studies (*n* = 23) [36, 49, 52, 53, 57, 68, 69, 73, 79, 80, 84, 85, 91, 95, 102, 121, 122, 126, 128, 131], pilot studies (*n* = 10) [55, 61, 75, 111, 118, 129, 130, 134–136], feasibility studies (*n* = 8) [35, 76, 78, 87, 88, 103, 109, 133], or validation studies (*n* = 6) [46, 50, 62, 72, 93, 100]. Less applied designs included randomized controlled trials (*n* = 3) [41, 65, 96], pre-poststudies (*n* = 3) [49, 66, 132], those developing outcomes (*n* = 3) [89, 113, 116], two case studies [71, 99], two mixed-methods studies [45, 90], one historic cohort [110], one study developing an intervention [70], and one post-hoc analysis of a randomized trial [77].

Among the included reports, the aim was to develop a new PROM (*n* = 2) [89, 113], to test a PROM (*n* = 13) [46, 50, 60–62, 67, 72, 90, 93, 100, 105, 109, 116], to evaluate an intervention with PROM (*n* = 21) [35, 41, 51, 65, 66, 70, 75, 76, 78, 79, 84, 87, 88, 96, 102, 103, 111, 129, 132, 133, 135], or to assess characteristics of a study population by using PROMs (*n* = 62) [36, 43–45, 47–49, 53–56, 59, 63, 64, 68, 69, 71, 73, 74, 77, 80–83, 85, 86, 91, 92, 94, 95, 97–99, 101, 104, 106–108, 110, 112, 114, 115, 117–128, 130, 131, 134, 136].

In 60 studies, only one PROM was used [36, 41–45, 47, 49, 51–55, 57–59, 63, 64, 66, 68, 71–73, 78–84, 86–90, 92, 94, 95, 97, 98, 105, 107, 109, 110, 112, 113, 116–118, 120, 121, 123–128, 130, 134, 136], while 21 studies used a combination of two PROMs [35, 50, 56, 60–62, 67, 69, 70, 74, 76, 85, 91, 93, 99–101, 103, 111, 122, 131], eight studies combined three PROMs [75, 102, 108, 114, 115, 119, 129, 135], and three studies combined four PROMs [46, 96, 104]. The remaining six studies combined five or more PROMs [48, 77], or had outcome measures to subsets of respondents or various numbers of PROMs applied to subsets of their study population [65, 106, 132, 133].

### Geographical spread among the included reports

**Fig. 2:**
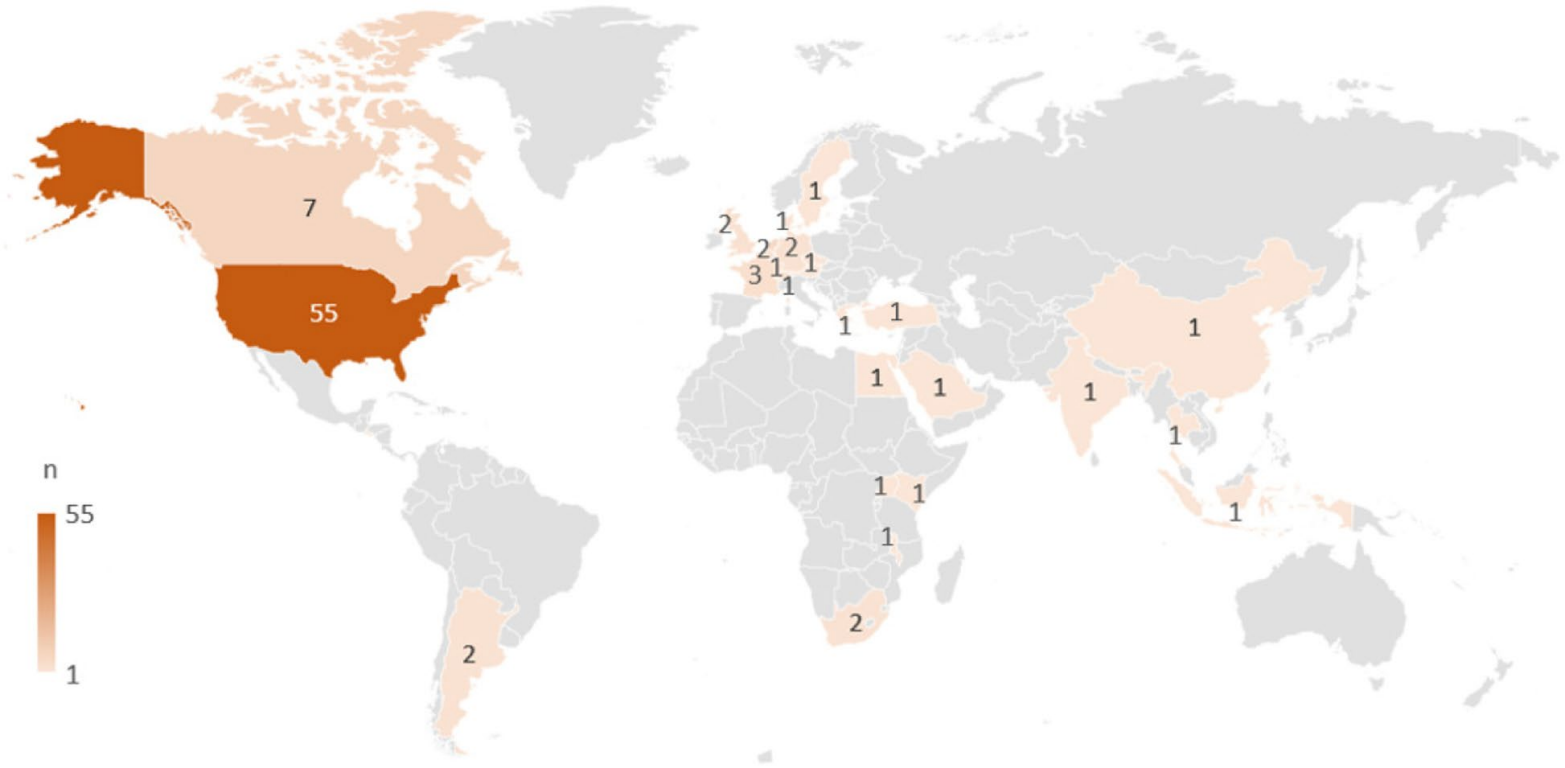
Included reports across the world

The included reports presented research conducted in 27 different countries across 5 continents (Fig. 2 and Supplementary file 4): North America (*n* = 55 studies, represented in *n* = 63 reports) [35, 36, 41, 45, 47, 51, 53, 58–60, 62–66, 72, 74–81, 83–86, 88, 89, 91, 95–97, 99, 101, 103–106, 111, 113–118, 121, 122, 126–136], Europe (*n* = 20 studies; *n* = 21 reports) [46, 49, 50, 52, 54, 55, 61, 67, 68, 70, 71, 73, 82, 87, 98, 107–109, 120, 124, 125], Asia (*n* = 9 reports; *n* = 8 studies) [42–44, 48, 93, 94, 110, 112, 119], Africa (*n* = 4 studies, *n* = 4 reports) [56, 57, 90, 92], and Central and South America (*n* = 3 studies and *n* = 3 reports) [100, 102, 108]. One report was a collaborative study between the Netherlands, Belgium, and Germany [107], another was a collaborative study between Germany and the Czech Republic [70], a third was a comparative study between Sweden and Argentina [108], and a fourth report was collaborative study between Kenya, Uganda, and South Africa [90].

### Development in publications on PROMs for children with LL/LT conditions

**Fig. 3:**
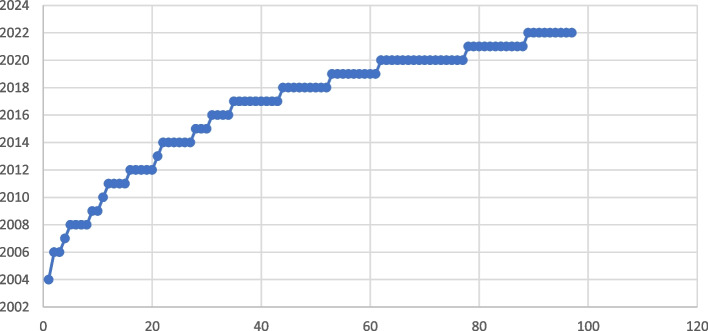
Reports published per year. Publication year is the vertical line whilst the number of publications is the horizontal line, and each blue dot represents a publication in this review

Based on the included reports eligible for review, there has been a marked increase in publications over the years (Fig. 3). There is an increasing number of publications per year, with slightly more in 2020.

## Results of the consultation exercise

Four consultations were conducted with three different stakeholder groups with interests in PPC (Table 5). Comments included acknowledgment of the importance and the clinical relevance, suggestions for the next step of the development of PROMs, and whether the results met the expectations of those in the audience or not. Sharing preliminary findings with peers increased the relevance of our findings for future research and clinical practice.

**Table 5:** Consultation exercise participants and feedback

Setting	Participants	Feedback
CHIP research network meeting, discussion led by the first author	Around 25 participants, comprising researchers, clinicians, and user representatives	Relatable findings Remain a need for outcomes besides quality of life Interest in the specific diagnoses Emphasize on the symptoms that are targeted in the outcomes Interesting with the associations between phenomena and outcome measure
National palliative care meeting, discussion led by the last author	Around 30 peers, most of whom worked in adult palliative care	Astonished by the high number of PROMs related to PPC, compared to the limited number of PROMs which are applied in clinical adult palliative care
National quality of life research network, discussion led by the first author	Around 40 peers, most of whom works in health research and higher education	Discussing the pros and cons of the scoping review methodology and how to handle the high number of identified reports Lack of psychometric properties among the extracted data, but consensus regarding our decision to firstly scope the identified PROMs and current research
National PPC meeting, poster presentation led by the first author	Around 200 peers, comprising mostly health professions, but also researchers and people with user experience	Acknowledging the relevance of PROMs to ensure patient-centered and family-centered care Experiences with PROMs included the use of visual reporting of pain

*CHIP* Children in Palliative Care, *PPC* Pediatric palliative care, *PROM* Patient-reported outcome measure

## Discussion

The aim of this scoping review was to obtain an overview of PROMs, including both patient-and proxy-reported outcome measures, used by and for CAYAs with LL/LT-conditions eligible for PPC in clinical and research settings. The increasing number of eligible reports during the past 5–10 years indicate an area of high research activity. We identified a total of 80 PROMs where the phenomena most frequently measured was QOL. Being an overarching concept in PPC [138], our review identified health-related, disease specific, and generic measures of QOL operationalized as an outcome measure in 21 of the included PROMs. Some of these PROMs on QOL were disease-specific, aiming at covering any disease-related symptoms and their effects on QOL [72]. These findings are in line with a previous review on HRQL instruments for children with LL/LT conditions [17].

The second most measured phenomena were about symptoms, either specific symptoms such as pain or combinations of symptoms, for example pain and anxiety. These findings are in accordance with existing evidence, which has shown a high number of outcome measures for the assessment of pain [139]. PROMs targeting specific symptoms or disease-specific instruments of QOL often focus on the limitations of a disease on functioning [140]. In the search for appropriate PROMs researchers and clinicians should be cognizant to selecting instruments that are positively worded and focus on well-being rather than ill-being. Disease-specific instruments are more responsive and may be more clinically useful than generic instruments, while generic instruments are valuable as they allow for comparison across patient populations. However, central aspects of a child’s life affecting their QOL, such as playing, being with friends, their ability to participate in school, or even their autonomy, have not been much reflected in the identified PROMs in this review. Acknowledging the importance of various PROMs in future studies is crucial and corresponds well with the developmentally appropriate symptom assessment tools that are among the top 20 research priorities in PPC [33].

As PPC has been described as the treatment of “total pain” [141], the ideal multidimensional PROM should encompass not only physical, but also psychological, social, existential, and practical matters [12]. This is supported by the study of Ribbers et al. [13] which identified six core outcome domains among parents of children with life-limiting conditions and neurological impairment: symptom control, respite and support, normalcy, security, empowerment, and coping with the disease. However, the complexity of composite outcome tools measuring several aspects might make them less useful in a clinical settings [84]. A clinically relevant and practical solution could be the application of an initial, single-item PROM indicating if there is a need for assessment with more comprehensive PROMs. An example is one brief question about whether the child is at peace [142], or the distress thermometer screening tool [133]. The short time needed to answer such brief PROMs contributes to their usefulness in clinical practice. Another way of reducing the number of PROM items could be to use a computerized assessment system (CAT), which has been previously used in children [143], or more responsive tools; where each next item presented to the respondent is based on the previous response. Such responsive tools have been applied in several of the studies in this review [47, 48, 53, 64, 69, 74, 78, 99, 101, 131, 135].

Overall, practical aspects were of little focus among the included papers. Of those reporting modes of delivery, paper, or web-based were equally frequent, while the time to complete the PROMs ranged from 45 s to 2 h. A well-described barrier concerning existing validated tools is the lengthy time required to complete a PROM [84]. The lack of focus on delivery mode and expected response time may be explained by the fact that children in PPC constitute a heterogeneous group; thus, it may be challenging to provide time estimates because children’s ability to self-report might vary. In addition, outcomes are often reported together, making it challenging to describe such details for each PROM. The study design and rationale of applying and reporting on a PROM might also provide some explanation to why details on the practicalities regarding PROMs was lacking. If the use of a PROM is to assess its feasibility in a clinical setting, the practicalities should be reported alongside the PROM characteristics. Thus, more focus on the practicalities of PROMs remains important for future research and in future reporting of PROM research.

The most frequently studied condition among the reviewed reports is childhood cancer, representing TfSL- patient group 1 [39]. This finding corresponds with previous reviews [12, 14, 15], although from a different methodological approach. A majority of the reports focused on CAYAs with cancer in the course of care pathway 2, i.e. during ongoing care, while a few (retrospective) reports focus on young cancer patients in care pathway 3 (end-of-life care). Two studies reported on the application of PROMs for CAYAs with cancer in care pathway 1 (at time of diagnosis) [85, 95]. It is not possible to conclude why there is a lack of PROMs reported in other patient groups. One reason might be that the field of PPC has developed from adult palliative care, which originated from adult oncology [144]. This could be a reason why CAYAs in TfSL- groups 2–4 have not been the focus of PROMs studies in PPC. It is noteworthy that CAYAs with neurological LL/LT conditions represent the group with the second highest prevalence within PPC in the Western world [145], yet the vast majority of studies on PROMs in PPC have not included these patients. Hence, there is a large potential to increase and improve the use of PROMs (including proxy-reporting) to better capture the needs and outcomes of CAYAs with progressive neurodegenerative or metabolic conditions [12, 61]. The misconception of PPC as exclusively end-of-life care may be another reason why some groups of patients have rarely been included in PROM research [146–148].

Proxy reporting is the only way to obtain patient-centered outcomes for most CAYAs in TfSL-group 3, for patients in the perinatal pathway and in end-of-life care. Issues related to proxy reporting is a complex matter [22, 112, 128] and far beyond the scope of this review. Several studies have found indications of discordance between patient and proxy reporting in PPC [128, 149]. Although previous evidence has suggested that validated tools for patient self-reporting are scarce in PPC [15], our review identified a high number of PROMs used in CAYAs eligible for PPC. Studies of PROMs for children and adolescents nearing the end of life have shown that self-reporting is possible, even under such severe circumstances, provided the use of appropriate modes of delivery (such as electronic devices) and flexibility for the patient to self-report [88, 136]. Furthermore, gamification has been on the rise in PPC and can be a well-suited method to allow children to self-report on individualized outcomes, such as through the electronic PROM called AquaScouts [70]. Thus, future research should explore the use of new administration modes such as pictures, drawings, or pictograms in combination with new technologies.

### Limitations

Despite efforts to conduct a comprehensive and systematic literature search, there remains a risk of missing relevant studies that should have been identified. We did not conduct reference searches among the included reports, and some papers may have been missed due to language restrictions. In addition, although the amount of gray literature on this subject is substantial, this was not included as our review was limited to the inclusion of reports (studies) published in peer-reviewed journals.

One aim of this scoping review was to find out if the identified PROMs have been developed specifically for CAYAs with LL/LT conditions, or if they are adapted versions of adult PROMs. This aim had to be abandoned because of a lack of reported details on PROM origination in the included reports, which was also noted in previous research on outcomes in PPC [15]. As a scoping review, this study did not examine methodological aspects of the included PROMs.

The included reports are from studies conducted in North America or Europe, which is in line with previous findings on the lack of geographical spread in PPC research [12]. Thus, the PROMs identified in this review are not immediately transferable to low- or middle-income countries, which is where 98% of CAYAs in need of PPC live [3].

Information about the PROMs in this review has been based on the included reports exclusively. We have not included data from any other reports or from the original versions of PROMs. This might preclude our impression regarding the included PROMs because we lack data on their development and validation because of weak reporting on PROM details in the included reports. Future research should specifically focus on the quality of the PROMs, including validity and reliability of the measures. Another limitation could be the fact that we did not include the terms patient-centered and family-centered in our search string. Instead, we used “proxy-reports” as PROMs. Lastly, we might have missed reports studying the social or existential elements of PPC, rather than the physical and psychological domains in which we have identified. Research investigating patient-reports according to the broad understanding and definition of PPC [1] is welcomed.

To categorize the diseases among the CAYAs of the included reports, we applied the TfSL categorization [24]. The TfSL groups are not prescriptive or definitive, and some diseases could be categorized under more than one category and / or a different category. Our categorization was done according to the TfSL based on our clinical experience and discussion among the researchers, but we acknowledge that others may categorize them differently. Lastly, we did not apply the revised categorization by Benini and colleagues [4], as it was published after our extraction and categorization was initiated.

## Conclusion

A broad range of phenomena relevant to CAYAs eligible for PPC are covered by the PROMs identified in this scoping review. So far, PROMs research in this population has focused on patients with cancer or organ failure living in high-income countries, mainly investigating physical phenomena. Despite an increasing activity in this field of research during recent years, the patient populations seem to be similar as in previous reviews. Future research should aim at exploring the usefulness of PROMs in CAYAs with other LL/LT conditions, including proxy-reported, patient- and family-centered outcomes. More research on PROMs related to all domains of PPC is welcomed.

### Supplementary Information

**Additional file 1.** Preferred Reporting Items for Systematic reviews and Meta-Analyses extension for Scoping Reviews (PRISMA-ScR) Checklist.

**Additional file 2: Supplementary file 2.** Holmen et al. Patient-reported outcome measures in children, adolescents, and young adults with palliative care needs - a scoping review.

**Additional file 3.** Search strategies.

**Additional file 4.** Extracted data Holmen PROMs.

**Additional file 5.** A complete list of identified PROMs.

## Data Availability

The datasets generated in the scoping searches will be made available upon reasonable request to the first author Heidi Holmen.

## Abbreviations

AMED: Allied and Complementary Medicine Database
APA: American Psychological Association
CAYA: Children, adolescents, and young adults
CHIP: Children in Palliative Care
CINAHL: Cumulative Index to Nursing and Allied Health Literature
EMBASE: Excerpta Medica database
HaPI: Health and Psychosocial Instruments
HCP: Healthcare personnel
HRQL: Health-related quality of life
LL/LT: Life-limiting or life-threatening
Medline: Medical Literature Analysis and Retrieval System Online
Ped-PRO-CTCAE: Pediatric Patient-Reported Outcomes version of the Common Terminology Criteria for Adverse Events
PPC: Pediatric palliative care
PRESS: Peer Review of Electronic Search Strategies
PRISMA ScR: Preferred Reporting Items for Systematic Reviews and Meta-Analyses-Scoping review extension
PREM: Patient-reported experience measure
PROM: Patient-reported outcome measure
PTSD: Post-traumatic stress disorder
QOL: Quality of life
WHO: World Health Organization
