# Supplementary material for: Patient-reported outcome measures in children, adolescents, and young adults with palliative care needs—a scoping review
Source: BMC Palliat Care. 2023 Oct 6;22:148. doi: 10.1186/s12904-023-01271-9 (PMC10557323; doi:10.1186/s12904-023-01271-9)
Supplement: Supplementary file 2 — Additional file 2: Supplementary file 2. Holmen et al. Patient-reported outcome measures in children, adolescents, and young adults with palliative care needs - a scoping review. [file 12904_2023_1271_MOESM2_ESM.docx]

Supplementary file 2. Holmen et al. Patient-reported outcome measures in children, adolescents, and young adults with palliative care needs - a scoping review

# Suppl 2. Deviations from the published protocol

## Protocol publication:

Holmen H, Winger A, Steindal SA, Castor C, Kvarme LG, Riiser K, Mariussen KL, Lee A. Patient-reported outcome measures in pediatric palliative care—a protocol for a scoping review. Systematic Reviews. 2021 Dec;10(1):1-6.

## Registration:

https://osf.io/yfch2/

**Table. Deviations from the scoping review protocol**

| Per protocol | Deviation from protocol |
| --- | --- |
| Title: PROM in PPC – a scoping review | We found a need to clarify the intention of the scoping review, and that the PROMs had to relevant for children eligible for palliative care, not only those already receiving palliative care. New title: Patient-reported outcome measures in children, adolescents and young adults with palliative care needs - a scoping review |
| Aim: To provide an overview of peer-reviewed studies on PROMs in PPC to map the nature of current research, to identify and summarize existing knowledge, and to present current knowledge gaps. | We found a need to clarify the intention of the scoping review, and that the PROMs had to relevant for children eligible for palliative care, not only those already receiving palliative care.  Clarified aim: to provide an overview of PROMs relevant for CAYAs living with LL/LT conditions eligible for pediatric palliative care (PPC). |
| Eligible studies will be transferred from Covidence to the qualitative data analysis software NVivo […]. | We did not use NVivo for text analysis, as the material was better suited for analysis using excel. |
| Any quantitative findings will be transformed to qualitative text […]. | There was no need to transform data from numbers to text. |
| Inspired by thematic synthesis developed by Thomas and Harden […], we aim to conduct a line-by-line coding and develop descriptive themes. As scoping reviews do not aim to provide a synthesis of findings, we will not generate any analytical themes to synthesize the data. | The number of relevant reports to include in our scoping exceeded our expectations. Thus, the material was better suited for descriptive and more numerical summary than a textual summary of descriptive themes. |
