# Supplementary material for: Patient-reported outcome measures in children, adolescents, and young adults with palliative care needs—a scoping review
Source: BMC Palliat Care. 2023 Oct 6;22:148. doi: 10.1186/s12904-023-01271-9 (PMC10557323; doi:10.1186/s12904-023-01271-9)
Supplement: Supplementary file 3 — Additional file 3. Search strategies. [file 12904_2023_1271_MOESM3_ESM.docx]

Search strategies

# Ovid MEDLINE(R) and Epub Ahead of Print, In-Process & Other Non-Indexed Citations and Daily

| 1 | Palliative Care/ |
| --- | --- |
| 2 | "Hospice and Palliative Care Nursing"/ |
| 3 | exp Terminal Care/ |
| 4 | Palliative Medicine/ |
| 5 | exp Advance Care Planning/ |
| 6 | Resuscitation Orders/ |
| 7 | "Right to Die"/ |
| 8 | Terminally ill/ |
| 9 | (palliative or palliate* or palliating).tw,kf. |
| 10 | (dying or (right adj2 die) or (die adj2 dignity)).tw,kf. |
| 11 | "supporti* care".tw,kf. |
| 12 | ((terminal* or "end stage*" or endstage* or "advanced stage*" or "late stage*") adj3 (disease* or ill* or care* or caring or treatment* or period* or nurs* or patient*)).tw,kf. |
| 13 | (eol or "end of life").tw,kf. |
| 14 | (("life limiting" or "life threatening") adj3 (disease* or condition* or illness*)).tw,kf. |
| 15 | (DNR or DNAR or DNI or ("do not" adj3 (intubat* or resuscitat*))).tw,kf. |
| 16 | "comfort measure*".tw,kf. |
| 17 | (advance*1 adj3 (plan*1 or planning or directive*)).tw,kf. |
| 18 | hospice*.tw,kf. |
| 19 | or/1-18 |
| 20 | exp *Child/ or exp *Infant/ or *Adolescent/ or *Young Adult/ |
| 21 | exp Intensive Care Units, Pediatric/ or (PICU* or NICU*).tw,kf. |
| 22 | exp Pediatrics/ or exp Pediatric Nursing/ or (pediatric* or paediatric* or peadiatric*).tw,kf. |
| 23 | (neonatal* or neo-natal* or neonate* or newborn* or new-born* or infant* or baby or babies or toddler* or child* or childhood or kid or kids or girl or girls or boy or boys or minors or underage* or under-age* or teen* or youth* or youngster* or adolescent* or adolescence or preadoles* or pre-adoles* or juvenil* or puber* or pubescen* or pre-puber* or prepuber* or prepubescen* or pre-pubescen* or (young adj2 (adult* or man or men or woman or women or person* or people))).tw,kf. |
| 24 | or/20-23 |
| 25 | 19 and 24 |
| 26 | Patient Reported Outcome Measures/ |
| 27 | exp Patient Outcome Assessment/ |
| 28 | Self Report/ |
| 29 | (PROM or PROMS or PREM or PREMS).tw,kf. |
| 30 | (selfreport* or patientreport* or ((self or patient* or proxy or proxies or family or families or caregiver* or parent* or mother* or father* or brother* or sister* or sibling*) adj3 report*)).tw,kf. |
| 31 | ((nurse* or therapist* or physiotherapist* or physician* or pediatrician* or paediatrician* or neonatologist* or cardiologist* or neurologist* or oncologist*) adj3 report*).tw,kf. |
| 32 | ("patient oriented" adj3 (outcome* or measure* or assessment*)).tw,kf. |
| 33 | (("patient centered" or "patient centred") adj3 (outcome* or measure* or assessment*)).tw,kf. |
| 34 | (patient* adj3 "outcome assessment*").tw,kf. |
| 35 | or/26-34 |
| 36 | 25 and 35 |
| 37 | limit 36 to (comment or editorial or letter) |
| 38 | 36 not 37 |
| 39 | limit 38 to (danish or english or german or norwegian or swedish) |

# AMED (Allied and Complementary Medicine)

| 1 | palliative care/ |
| --- | --- |
| 2 | exp terminal care/ |
| 3 | palliative medicine/ |
| 4 | advance directives/ |
| 5 | right to die/ |
| 6 | terminal illness/ |
| 7 | (palliative or palliate* or palliating).tw. |
| 8 | (dying or (right adj2 die) or (die adj2 dignity)).tw. |
| 9 | "supporti* care".tw. |
| 10 | ((terminal* or "end stage*" or endstage* or "advanced stage*" or "late stage*") adj3 (disease* or ill* or care* or caring or treatment* or period* or nurs* or patient*)).tw. |
| 11 | (eol or "end of life").tw. |
| 12 | (("life limiting" or "life threatening") adj3 (disease* or condition* or illness*)).tw. |
| 13 | resuscitation orders/ or (DNR or DNAR or DNI or ("do not" adj3 (intubat* or resuscitat*))).tw. |
| 14 | "comfort measure*".tw. |
| 15 | (advance*1 adj3 (plan*1 or planning or directive*)).tw. |
| 16 | hospice*.tw. |
| 17 | or/1-16 |
| 18 | exp adolescent/ or exp child/ or exp infant/ |
| 19 | (PICU* or NICU*).tw. |
| 20 | pediatrics/ or (pediatric* or paediatric* or peadiatric*).tw. |
| 21 | (neonatal* or neo-natal* or neonate* or newborn* or new-born* or infant* or baby or babies or toddler* or child* or childhood or kid or kids or girl or girls or boy or boys or minors or underage* or under-age* or teen* or youth* or youngster* or adolescent* or adolescence or preadoles* or pre-adoles* or juvenil* or puber* or pubescen* or pre-puber* or prepuber* or prepubescen* or pre-pubescen* or (young adj2 (adult* or man or men or woman or women or person* or people))).tw. |
| 22 | or/18-21 |
| 23 | 17 and 22 |
| 24 | (PROM or PROMS or PREM or PREMS).tw. |
| 25 | (selfreport* or patientreport* or ((self or patient* or proxy or proxies or family or families or caregiver* or parent* or mother* or father* or brother* or sister* or sibling*) adj3 report*)).tw. |
| 26 | ((nurse* or therapist* or physiotherapist* or physician* or pediatrician* or paediatrician* or neonatologist* or cardiologist* or neurologist* or oncologist*) adj3 report*).tw. |
| 27 | ("patient oriented" adj3 (outcome* or measure* or assessment*)).tw. |
| 28 | (("patient centered" or "patient centred") adj3 (outcome* or measure* or assessment*)).tw. |
| 29 | (patient* adj3 "outcome assessment*").tw. |
| 30 | or/24-29 |
| 31 | 23 and 30 |
| 32 | limit 31 to (commentary or editorial or letter) |
| 33 | limit 31 to (danish or english or german or norwegian or swedish) |

# CINAHL

| S37 | S35 not S36 | 626 |
| --- | --- | --- |
| S36 | S24 AND S33  Limiters - Language: Danish, English, German, Norwegian, Swedish; Publication Type: Commentary, Editorial, Letter | 8 |
| S35 | S24 AND S33  Limiters - Language: Danish, English, German, Norwegian, Swedish Search modes - Boolean/Phrase | 634 |
| S34 | S24 AND S33 | 638 |
| S33 | S25 OR S26 OR S27 OR S28 OR S29 OR S30 OR S31 OR S32 | 211,887 |
| S32 | (patient* N2 "outcome assessment*") | 192 |
| S31 | (("patient centered" or "patient centred") N2 (outcome* or measure* or assessment*)) | 1,965 |
| S30 | ("patient oriented" N2 (outcome* or measure* or assessment*)) | 302 |
| S29 | ((nurse* or therapist* or physiotherapist* or physician* or pediatrician* or paediatrician* or neonatologist* or cardiologist* or neurologist* or oncologist*) N2 report*) | 12,020 |
| S28 | (selfreport* or patientreport* or ((self or patient* or proxy or proxies or family or families or caregiver* or parent* or mother* or father* or brother* or sister* or sibling*) N2 report*)) | 200,505 |
| S27 | (PROM or PROMS or PREM or PREMS) | 2,015 |
| S26 | (MH "Self Report+") | 74,028 |
| S25 | (MH "Patient-Reported Outcomes+") | 3,006 |
| S24 | S18 AND S23 | 13,236 |
| S23 | S19 OR S20 OR S21 OR S22 | 947,059 |
| S22 | TI (neonatal* or neo-natal* or neonate* or newborn* or new-born* or infant* or baby or babies or toddler* or child* or childhood or kid or kids or girl or girls or boy or boys or minors or underage* or under-age* or teen* or youth* or youngster* or adolescent* or adolescence or preadoles* or pre-adoles* or juvenil* or puber* or pubescen* or pre-puber* or prepuber* or prepubescen* or pre-pubescen* or (young N1(adult* or man or men or woman or women or person* or people))) or AB (neonatal* or neo-natal* or neonate* or newborn* or new-born* or infant* or baby or babies or toddler* or child* or childhood or kid or kids or girl or girls or boy or boys or minors or underage* or under-age* or teen* or youth* or youngster* or adolescent* or adolescence or preadoles* or pre-adoles* or juvenil* or puber* or pubescen* or pre-puber* or prepuber* or prepubescen* or pre-pubescen* or (young N1 (adult* or man or men or woman or women or person* or people))) | 862,704 |
| S21 | (MH "Pediatrics+") or (MH "Pediatric Nursing+") or (pediatric* or paediatric* or peadiatric*) | 202,169 |
| S20 | (MH "Intensive Care Units, Pediatric+") or (PICU* or NICU*) | 23,843 |
| S19 | (MM "Child+") OR (MM "Young Adult") OR (MM "Adolescence+") | 41,047 |
| S18 | S1 OR S2 OR S3 OR S4 OR S5 OR S6 OR S7 OR S8 OR S9 OR S10 OR S11 OR S12 OR S13 OR S14 OR S15 OR S16 OR S17 | 131,463 |
| S17 | hospice* | 20,990 |
| S16 | (advance* N2 (plan or plans or planning or directive*)) | 10,733 |
| S15 | "comfort measure*" | 337 |
| S14 | (MH "Resuscitation Orders") or (DNR or DNAR or DNI or ("do not" N2 (intubat* or resuscitat*))) | 4,116 |
| S13 | (("life limiting" or "life threatening") N2 (disease* or condition* or illness*)) | 6,885 |
| S12 | (eol or "end of life") | 20,807 |
| S11 | ((terminal* or "end stage*" or endstage* or "advanced stage*" or "late stage*") N2 (disease* or ill* or care* or caring or treatment* or period* or nurs* or patient*)) | 48,013 |
| S10 | "supporti* care" | 6,220 |
| S9 | (dying or (right N1 die) or (die N1 dignity)) | 18,314 |
| S8 | (palliative or palliate* or palliating) | 53,427 |
| S7 | (MH "Terminally Ill Patients+") | 12,064 |
| S6 | (MH "Right to Die") | 1,664 |
| S5 | (MH "Advance Care Planning") | 3,782 |
| S4 | (MH "Hospice Care") | 9,084 |
| S3 | (MH "Terminal Care+") | 66,905 |
| S2 | (MH "Hospice and Palliative Nursing") | 5,089 |
| S1 | (MH "Palliative Care") | 37,219 |

# Embase

| 1 | exp palliative therapy/ |
| --- | --- |
| 2 | palliative nursing/ |
| 3 | exp terminal care/ |
| 4 | right to die/ |
| 5 | exp terminally ill patient/ |
| 6 | (palliative or palliate* or palliating).tw,kw. |
| 7 | (dying or (right adj2 die) or (die adj2 dignity)).tw,kw. |
| 8 | "supporti* care".tw,kw. |
| 9 | ((terminal* or "end stage*" or endstage* or "advanced stage*" or "late stage*") adj3 (disease* or ill* or care* or caring or treatment* or period* or nurs* or patient*)).tw,kw. |
| 10 | (eol or "end of life").tw,kw. |
| 11 | (("life limiting" or "life threatening") adj3 (disease* or condition* or illness*)).tw,kw. |
| 12 | (DNR or DNAR or DNI or ("do not" adj3 (intubat* or resuscitat*))).tw,kw. |
| 13 | "comfort measure*".tw,kw. |
| 14 | (advance*1 adj3 (plan*1 or planning or directive*)).tw,kw. |
| 15 | hospice*.tw,kw. |
| 16 | or/1-15 |
| 17 | exp *child/ or exp *infant/ or exp *adolescent/ or exp *young adult/ |
| 18 | neonatal intensive care unit/ or pediatric intensive care unit/ or (PICU* or NICU*).tw,kw. |
| 19 | exp pediatrics/ or exp pediatric nursing/ or (pediatric* or paediatric* or peadiatric*).tw,kw. |
| 20 | (neonatal* or neo-natal* or neonate* or newborn* or new-born* or infant* or baby or babies or toddler* or child* or childhood or kid or kids or girl or girls or boy or boys or minors or underage* or under-age* or teen* or youth* or youngster* or adolescent* or adolescence or preadoles* or pre-adoles* or juvenil* or puber* or pubescen* or pre-puber* or prepuber* or prepubescen* or pre-pubescen* or (young adj2 (adult* or man or men or woman or women or person* or people))).tw,kw. |
| 21 | or/17-20 |
| 22 | 16 and 21 |
| 23 | exp patient-reported outcome/ |
| 24 | self report/ |
| 25 | (PROM or PROMS or PREM or PREMS).tw,kw. |
| 26 | (selfreport* or patientreport* or ((self or patient* or proxy or proxies or family or families or caregiver* or parent* or mother* or father* or brother* or sister* or sibling*) adj3 report*)).tw,kw. |
| 27 | ((nurse* or therapist* or physiotherapist* or physician* or pediatrician* or paediatrician* or neonatologist* or cardiologist* or neurologist* or oncologist*) adj3 report*).tw,kw. |
| 28 | ("patient oriented" adj3 (outcome* or measure* or assessment*)).tw,kw. |
| 29 | (("patient centered" or "patient centred") adj3 (outcome* or measure* or assessment*)).tw,kw. |
| 30 | (patient* adj3 "outcome assessment*").tw,kw. |
| 31 | or/23-30 |
| 32 | 22 and 31 |
| 33 | limit 32 to (conference abstract or conference paper or "conference review" or editorial or letter) |
| 34 | 32 not 33 |
| 35 | limit 34 to (danish or english or german or norwegian or swedish) |

# APA PsycInfo

| 1 | palliative care/ |
| --- | --- |
| 2 | hospice/ |
| 3 | advance directives/ |
| 4 | terminally ill patients/ |
| 5 | (palliative or palliate* or palliating).tw. |
| 6 | (dying or (right adj2 die) or (die adj2 dignity)).tw. |
| 7 | "supporti* care".tw. |
| 8 | ((terminal* or "end stage*" or endstage* or "advanced stage*" or "late stage*") adj3 (disease* or ill* or care* or caring or treatment* or period* or nurs* or patient*)).tw. |
| 9 | (eol or "end of life").tw. |
| 10 | (("life limiting" or "life threatening") adj3 (disease* or condition* or illness*)).tw. |
| 11 | (DNR or DNAR or DNI or ("do not" adj3 (intubat* or resuscitat*))).tw. |
| 12 | "comfort measure*".tw. |
| 13 | (advance*1 adj3 (plan*1 or planning or directive*)).tw. |
| 14 | hospice*.tw. |
| 15 | or/1-14 |
| 16 | exp neonatal intensive care/ or (PICU* or NICU*).tw. |
| 17 | exp pediatrics/ or (pediatric* or paediatric* or peadiatric*).tw. |
| 18 | (neonatal* or neo-natal* or neonate* or newborn* or new-born* or infant* or baby or babies or toddler* or child* or childhood or kid or kids or girl or girls or boy or boys or minors or underage* or under-age* or teen* or youth* or youngster* or adolescent* or adolescence or preadoles* or pre-adoles* or juvenil* or puber* or pubescen* or pre-puber* or prepuber* or prepubescen* or pre-pubescen* or (young adj2 (adult* or man or men or woman or women or person* or people))).tw. |
| 19 | or/16-18 |
| 20 | 15 and 19 |
| 21 | patient reported outcome measures/ |
| 22 | self-report/ |
| 23 | (PROM or PROMS or PREM or PREMS).tw. |
| 24 | (selfreport* or patientreport* or ((self or patient* or proxy or proxies or family or families or caregiver* or parent* or mother* or father* or brother* or sister* or sibling*) adj3 report*)).tw. |
| 25 | ((nurse* or therapist* or physiotherapist* or physician* or pediatrician* or paediatrician* or neonatologist* or cardiologist* or neurologist* or oncologist*) adj3 report*).tw. |
| 26 | ("patient oriented" adj3 (outcome* or measure* or assessment*)).tw. |
| 27 | (("patient centered" or "patient centred") adj3 (outcome* or measure* or assessment*)).tw. |
| 28 | (patient* adj3 "outcome assessment*").tw. |
| 29 | or/21-28 |
| 30 | 20 and 29 |
| 31 | limit 30 to ("comment/reply" or editorial or letter) |
| 32 | 30 not 31 |
| 33 | limit 32 to (danish or english or german or norwegian or swedish) |

# Health and Psychosocial Instruments (HaPI)

1. “palliative care”.mp.

2. (Hospice and palliative nusing).mp.

3. terminal care.mp.

4. Hospice care.mp.

5. Advance care planning.mp.

6. right to die.mp.

7. Terminal ill patients.mp.

8. (palliative or palliate* or palliating).mp.

9. End of life.mp.

10. 1 or 2 or 3 or 4 or 5 or 6 or 7 or 8 or 9

11. child.mp.

12. young adult.mp.

13. picu.mp.

14. nicu.mp.

15. pediatric nursing.mp.

16. paediatric.mp.

17. pediatric.mp.

18. 11 or 12 or 13 or 14 or 15 or 16 or 17

19. patient reported outcome.mp.

20. self-report.mp.

21. PROM.mp.

22. PREM.mp.

23. PRO.mp.

24. patient reported outcome measure.mp.

25. 19 or 20 or 21 or 22 or 23 or 24

26. 10 and 18 and 25 (generated no findings/ n=0)

27 10 and 18
