# Supplementary material for: Patient-reported outcome measures in children, adolescents, and young adults with palliative care needs—a scoping review
Source: BMC Palliat Care. 2023 Oct 6;22:148. doi: 10.1186/s12904-023-01271-9 (PMC10557323; doi:10.1186/s12904-023-01271-9)
Supplement: Supplementary file 4 — Additional file 4. Extracted data Holmen PROMs. [file 12904_2023_1271_MOESM4_ESM.docx]

| **References, alphabetically** | **Aim of the study** | **Study design** | **Patient group (TfSL)**^A^ | **Care Pathway (TfSL)**^B^ | **Participants** | **PROM details** |
| --- | --- | --- | --- | --- | --- | --- |
| *Author, year, country* | *As reported by the authors* |  |  |  | *Number, age, and diagnosis reported in the study* | *No. of PROMs and characteristics of each* |
| (Akard et al., 2020), USA | To present results that examine the impact of the web-based legacy intervention on quality of life (QOL) among children with relapsed or refractory cancer. | Randomized controlled trial | 1 | 2 | 150 children and 150 parents, inclusion criteria for age 7-17 years, mean age of children 10.4 years, cancer. | 1 PROM.  Pediatric Quality of Life Inventory^TM^ (PedsQL) Cancer Module self-report and proxy report, measuring health related quality of life (**HRQL)** in children. Disease-specific. Versions for 5–7, 8–12, and 13–17 years + proxy of children from 2-17 years. |
| (Alhusaini et al., 2019), Saudi Arabia | To record pediatric end stage renal disease (ESRD) patients’ QOL in relation to peritoneal dialysis (PD) and hemodialysis (HD). | Cross-sectional study | 1 | 2 | 23 children and 23 parents, inclusion criteria for age 2-18 years, mean age of children not reported (NR), end stage renal disease ESRD. | 1 PROM.  PedsQL 3.0 ESRD Module questionnaire measuring **HRQL** in children. Child self-reports and proxy-reports Disease-specific. |
| (Andriastuti, Halim, Kusrini, & Bangun, 2020), Indonesia | To describe the use of Pediatric Palliative Screening Scale (PaPaS Scale) and its depiction to the QOL of children with malignancies. | Cross-sectional study | 1 | 2 | 60 children and NR parents, inclusion criteria for age 2-18 years, mean age of children NR, cancer. | 1 PROM.  PedsQL cancer module 3.0 measuring **HRQL** in children. Disease-specific. |
| (Baek et al., 2018), Korea | To investigate the impact of pediatric ESRD on parents, based on the PedsQL Family Impact Module (FIM), and the relationship to the QOL of pediatric ESRD patients measured by PedsQL 3.0 ESRD module. | Cross-sectional study | 1 | 2 | 79 children and 158 parents, inclusion criteria for age 8-18 years, mean age of children 13.3 years, kidney transplants and ESRD. | 1 PROM.  PedsQL ESRD module measuring **HRQL** in children. Disease-specific. |
| (Baughcum et al., 2020), USA | To examine parent perceptions of their infant's end-of-life (EOL) experience (e.g., symptom burden and suffering) and satisfaction with care in the neonatal intensive care unit. | Mixed-methods study | Mixed | 3 | 40 infants and 67 parents, inclusion criteria for age infant, mean age of children NR, with various diagnosis (Cause of death Congenital, Respiratory, Cardiac, Other/not specified). | 1 PROM.  Study specific instrument based on the Memorial Symptom Assessment Scale to measure **Perceptions of infant symptoms and suffering.** Generic. |
| (Behan et al., 2019) United Kingdom | To assess the psychometric performance of QOL primary ciliary dyskinesia child, adolescent, and parent-proxy versions in terms of reliability and validity across cross-cultural settings and caring for patients with this rare disease. | Validation study | 2 | 2 | 156 children and 68 parents, inclusion criteria for age 6-17 years, mean age of children 12.7 years, with primary ciliary dyskinesia. | 4 PROMs  Quality of life for primary ciliary dyskinesia (QOL-PCD) measuring **disease-specific, HRQL**. Disease-specific. Prototypes of the QOL-PCD measures for children (aged 6-12 years), adolescents (aged 13-17 years), and parent-proxy (child 6-12 years).  PedsQL assessing **HRQL.** Generic.  COPD assessment test (CAT) assessing **respiratory specific QOL**.  Sinus and Nasal Quality of Life Survey (SN-5) assessing **QOL** related to nasal problems. |
| (Boyden et al., 2022) USA. Same study as Feudtner et al. 2021 | To examine the associations among parent psychological distress, parent-reported patient symptoms, and financial difficulty, seeking to determine the relative association of financial difficulty and of patient symptoms to parent psychological distress. | Cross-sectional study | Mixed | 2 | 532 children and 601 parents. Inclusion criteria for age 0-30 years, mean age of children 7.1 years with various diagnosis. | 1 PROM.  Pediatric Quality of Life and Evaluation of Symptoms Technology (PQ) Memorial Symptom Assessment Scale (PQ-MSAS) assessing child **symptoms**. Disease-specific. Previously adapted to a pediatric population. |
| (Cheng, Yuan, Wang, & Stinson, 2022) China | To investigate the prevalence, intensity, interference, and management of pain reported by Chinese children during cancer treatment and explore the predictors of pain interference. | Cross-sectional study | 1 | 2 | 187 children, inclusion criteria for age 8-17 years, mean age of children 10.8 years, cancer. | 7 PROMs.  Study specific items assessing **pain management and alleviation** over past 7 days  Patient Reported Outcomes Measurement Information System (PROMIS) **pain intensity.**  PROMIS **pain interference.**  PROMIS Pediatric Anger assessing co-occurring symptoms  PROMIS Anxiety assessing co-occurring symptoms  PROMIS Depressive Symptoms assessing co-occurring symptoms  PROMIS Fatigue assessing co-occurring symptoms  All assessments were self-reported by the child. |
| (Clavé et al., 2019) France | To describe the QOL of adolescents initiating hemodialysis treatment compared to French age- and sex-matched population; to investigate factors affecting their QOL; and to assess coping strategies and their impact on QOL. | Prospective study | 1 | 2 | 32 children, inclusion criteria for age 11-17 years, mean age of children 13.9 years, chronic renal insufficiency (CRI), chronic renal failure (CRF) or ESRD. | 1 PROM  “Vécu et Santé Perçue de l’Adolescent et l’Enfant” (VSP-A) questionnaire assessing **QOL** of adolescents, version for 11- to 17-year-olds. Generic. |
| (De Bruyne et al., 2022) Belgium | To translate the original version of the chronic kidney disease (CKD)-specific PedsQL™ 3.0 End Stage Renal Disease Module into a Dutch version and to evaluate its validity and reliability. | Validation study | 1 | 2 | 116 children and 62 parents, inclusion criteria for age NR, mean age of children 12.6 years with chronic kidney disease. | 2 PROMs.  PedsQL™ 3.0 ESRD Module measuring **HRQL** in children.  PedsQL™ 4.0 Generic Core Scale measuring **HRQL** in children. |
| (DeCourcey, Silverman, Oladunjoye, & Wolfe, 2019) USA | To describe the advanced care planning (ACP) communication priorities of bereaved parents of children, adolescents, and young adults with complex chronic conditions and to determine whether access to ACP influences parental preparedness for their child’s last days of life, ability to plan their child’s location of death, perceived child suffering and QOL at EOL, and parental decisional regret. | Cross-sectional study | Mixed | 3 | 114 parents on behalf for deceased children related to various diagnosis (CNS progressive, static encephalopathy, congenital chromosomal, neuromuscular, pulmonary). | 1 measure with PRO-items  Study specific measure with 183 items on parental perspectives on multiple domains regarding the care of children, adolescents, and young adults with complex chronic conditions - **perceived child suffering** as reported by parent-proxy**.** Generic. |
| (Derridj et al., 2022) France | To identify subgroups with a congenital heart defect (CHD) at risk of HRQL impairment at 8 years of age according to their medical and surgical management. | Prospective cohort study | 1 | 2 | 751 children and 751 parents, inclusion criteria for age 0-1 years, mean age of children at follow-up 8 years, with congenital heart defect. | 1 PROM  PedsQL 4.0 Generic Core Scales (self-report and proxy-report) assessing **HRQL**. |
| (Dobrozsi, Yan, Hoffmann, & Panepinto, 2017) USA | To measure patient-reported outcomes (PROs) in children during the initial 6 months of therapy to characterize function and explore factors associated with function including type of cancer, intensity of therapy, age, and gender. | Prospective cohort study | 1 | 1 | 40 children and 38 parents, inclusion criteria for age 5-21 years, mean age of children 11.7 years, cancer. | 1 PROM with multiple foci  PROMIS profile assessing patient **mobility, fatigue, pain interference, peer relationships, anxiety, and depressive symptoms**. Children aged 8 and older completed self-report PROs, and parents of all participants ages 5–21 completed proxy PROs. Generic. |
| (Dotis et al., 2016) Greece | To record the HRQL of children in Greece with CKD on peritoneal dialysis (PD) or who have had a renal transplant. | Cross-sectional study | 1 | 2 | 55 children and 45 parents, inclusion criteria for age 8-18 years, mean age of children 13.14 years, chronic kidney disease. | 1 PROM  Kidscreen-52, Greek version, measuring **HRQL** in healthy and chronically ill children and adolescents aged 8-18 years. Self-report and proxy report. Generic. |
| (Eijsermans, Creemers, Helders, & Schroder, 2004) Netherlands | To describe the motor skills, exercise tolerance, and health-related quality of life in children with ESRD. | Pilot study | 1 | 2 | 10 children, inclusion criteria for age 7-16 years, mean age of children 12.3, with chronic renal insufficiency (CRI), chronic renal failure (CRF) or end-stage renal disease (ESRD). | 1 PROM  TNO-AZL Questionnaires for Children's Health-Related Quality of Life (TACQOL), assessing **HRQL** in children with chronic disease, 7 domains. Self-report by the children. Generic. |
| (Ellis et al., 2021) Malawi | To longitudinally measure HRQL among pediatric lymphoma patients in Malawi to determine change throughout cancer treatment and association with survival | Prospective cohort study | 1 | 2 | 75 children and NR number of parents, inclusion criteria for age 5-18 years, mean age of children 10.0 years, cancer | 1 PROM  Pediatric PROMIS-25 PROFILE in Chichewa assessing **QOL**. For children ≥8 years old, the Chichewa version was administered directly to the child. The parent-proxy version was used for children 5–7 years old, cognitively impaired, or too ill to complete the questionnaire themselves. Generic. |
| (El Shafei, Soliman Hegazy, Fadel, & Nagy, 2018) Egypt | To assess QOL among children with ESRD either undergoing hemodialysis or had renal transplantation therapy and comparing it with healthy controls. | Cross-sectional study | 1 | 2 | 141 children and NR number of parents, inclusion criteria for age 5-18 years, mean age of children 11.9 years, kidney transplants and ESRD. | 2 PROMs  PedsQL v 4, measuring **HRQL** (four domains: physical domain, emotional domain, social domain, and school domain). parent and child reports. Generic.  PedsQL ESRD **disease specific HRQL** parent and child reports. |
| (Feudtner et al., 2021) USA. Same study as Boyden et al. 2022 | To provide a detailed description of the symptoms among patients receiving pediatric palliative care based on parental report via a validated, structured symptom assessment measure. | Cross-sectional study | Mixed | 2 | 501 children and 501 parents, inclusion criteria for age 0-30 years, mean age of children 4.1, various diagnosis. | 1 PROM.  PediQuest Memorial Symptom Assessment scale (PQ-MSAS) assessing **symptoms** in children, adapted to a pediatric population. Disease-specific. |
| (Fladeboe et al., 2021) USA | To determine the feasibility and acceptability of delivering an established resilience-coaching program, and integrating ACP into that program, among adolescent and young adults with advanced cancer. | Feasibility study | 1 | 2 | 26 children, inclusion criteria for age 12-24 years, mean age of children 16.0 years, cancer. | 2 PROMs.  The Hospital Anxiety and Depression Scale (HADS) assessing **depression symptoms and anxiety symptoms**. Generic.  Snyder **hope** scale assessing hope. Generic. |
| (Fortney et al., 2020) USA, A. Same study as Fortney et al. 2020 B. | To explore associations between nurse perceptions of infant well-being and self-reported distress. | Prospective longitudinal study | mixed | 0 | 78 infants and 237 healthcare personnel, inclusion criteria for age infants 23 weeks of gestation or more, mean age of children NR, various diagnosis (at least one potentially life threatening or life-limiting condition). | 1 PROM.  The Nurse Perceptions of Infant Well-Being Survey (developed for this study), assessing nurses’ perceptions of infant **suffering, symptoms** and current **QOL** on a 5-point Likert scale. Developed for proxy report. Generic. |
| (Fortney, Sealschott, & Pickler, 2020) USA, B. Same study as Fortney et al. 2020 A | To evaluate the use of the COMFORT Behavior (COMFORT-B) Scale as a measure of pain and distress in infants diagnosed with life-threatening or life-limiting illnesses in the neonatal intensive care unit (NICU) in comparison with the nurse-documented Neonatal Pain, Agitation and Sedation Scale (N-PASS); the infant’s Technology Dependence Scale (TDS); and the mother’s report of total perceived symptom (TPS) scores. | Prospective longitudinal study | Mixed | 0 | 78 infants and 76 parents, involved healthcare personnel NR, inclusion criteria for age ≤27 weeks of gestation, and mean age of children NR, various diagnosis (at least one potentially life threatening or life limiting (LT or LL) condition). | 2 PROMs.  COMFORT-B Scale is an adaptation of the COMFORT Scale, a behavioral assessment of **pain and distress**. Generic.  Nurse-documented Neonatal Pain, Agitation and Sedation Scale (N-PASS) is a noninvasive assessment tool of both acute and prolonged **pain, agitation, and sedation** in postoperative and mechanically ventilated infants in the NICU, including those born preterm. Generic. |
| (Friedel et al., 2020) Belgium | To assess the face and content validity, acceptability, and feasibility of a French version of the Children’s Palliative Outcome Scale (CPOS). | Pilot study, qualitative | mixed | 2 | 14 children, 19 parents and 9 healthcare personnel, inclusion criteria for age 8-18 years and mean age of children NR, various diagnosis (not specified other than oncological and neurological). | 2 PROMs  Children's palliative outcome scale (CPOS) assesses **palliative outcomes**. An instrument combining self-report (child) and proxy (parents) reports regarding health status, HRQOL, and QOL. It covers four dimensions: physical and psychological symptoms, information received, advance care planning, and self-efficacy. Generic.  Scheduled evaluation of individual quality of life (SEIQoL) measuring **QOL**. Developed for adults but adapted and validated for a pediatric population. Generic. |
| (Goldstein et al., 2008) USA | To investigate the feasibility, reliability, and validity of the PedsQL 3.0 ESRD Module and the PedsQL 4.0 Generic Core Scales at 4 pediatric ESRD centers. | Validity study | 1 | 2 | 193 children and 190 parents, inclusion criteria for age 5-18 years, mean age of children 13.6 years, ESRD. | 2 PROMs.  PedsQL 3.0 ESRD Module developed to yield detailed information for the specific factors that impact on **HRQL** in children with ESRD, 5-18 y (self-report), 2-4 y (proxy report). Disease-specific.  PedsQL 4.0 Core Scales measuring **HRQL** in children. Generic. |
| (Goldstein et al., 2009) USA | To investigate the differences in reported ESRD-specific HRQL across renal transplant and maintenance dialysis modalities utilizing the PedsQL ESRD Module. | Cross-sectional study | 1 | 2 | 186 children and 180 parents, inclusion criteria for age 2-18 years, mean age of children 13.4 years, ESRD. | 1 PROM.  PedsQL 3.0 ESRD Module assessing **HRQL** in children with ESRD, 5-18 y (self-report), 2-4 y (proxy report). Disease-specific. |
| (Grossoehme et al., 2020) USA | To model the association of spiritual and religious constructs with patient-reported outcomes of anxiety, depressive symptoms, fatigue, and pain interference. | Cross-sectional study | 1 | 2 | 126 children, inclusion criteria for age 14-21 years, mean age of children 16.9 years, cancer. | 4 PROMs.  PROMIS pediatric Anxiety. Generic  PROMIS pediatric Depressive symptoms. Generic  PROMIS pediatric Fatigue. Generic  PROMIS pediatric Pain interference. Generic |
| (Haller et al., 2019) USA | To employ a heat map analysis in order to understand the individual impact of treatment in this diverse population (MPS VII/Sly syndrome). | Randomized controlled trial | 3 | 2 | 12 children, inclusion criteria for age 5-35 years, mean age of children 15.4 years, with Sly syndrome mucopolysaccharidosis (MPS) VII. | 2 per child, 3 in the study due to age specific PROMs.  PROMIS health assessment questionnaire (**pain**) or Childhood health assessment questionnaire **pain** (CHAQ pain) depending on the age of the subject, assessing HRQOL pain dimension. Generic.  PedsQL multidimensional **fatigue** scale, assessing sleep/rest fatigue. Generic. |
| (Hays et al., 2006) USA | To report the effects of the program as measured by changes in family satisfaction and HRQL of life among the patients who participated. | Pre - posttest design | mixed | 2 + 3 | 41 children and number of parents NR, inclusion criteria for age 0-21 years, mean age of children 11.3 years, various diagnosis (cancer, progressive neurologic disorder, CNS damage, cystic fibrosis, congenital, metabolic, cardiac). | 1 PROM.  PedsQL 4.0 measuring **HRQL**. 5-18 y (self-report), 2-18 y (proxy report). Generic. |
| (Heath, Norman, Christian, & Watson, 2017) United Kingdom | To evaluate a generic and renal-specific self-report QOL scale, assess children’s attitudes towards living with CKD and propose an appropriate tool for future individual clinical use or departmental audit. | Cross-sectional study | 1 | 2 | 71 children, inclusion criteria for age 6-18 years, mean age of children 13.6 years, chronic kidney disease. | 2 PROMs  Generic Children's QoL Measure (GCQ) is a generic discrepancy measure of **psychosocial** **QOL**, developed to allow comparison between chronically ill children and the general child population. Generic.  PedsQL 3.0 ESRD module assessing the **HRQL** of children and adolescents with ESRD, 5-18 years. Disease-specific. |
| (Heye et al., 2019) Switzerland | To assess the impact of congenital heart disease (CHD) on daily life in preschoolers with single-ventricle CHD, and to identify determinants of HRQL. | Prospective cohort | 1 | 2 | 46 children and number of parents NR, inclusion criteria for age 0-4 years, mean age of children 3.1 years, CHD. | 1 PROM.  Pre-school Pediatric Cardiac Quality of Life Inventory assessing **HRQL** measuring frequency of disease-related problems and severity of negative emotions about these, by proxy report. |
| (Hinds et al., 2020) USA | To identify groups of children and adolescents reporting adverse events (AEs) using the Pediatric Patient-Reported Outcomes version of the Common Terminology Criteria for Adverse Events (Ped-PRO-CTCAE), to determine whether demographic and clinical characteristics predict AE group membership, to examine whether AE group membership was related to the distal outcome of psychological stress. | Prospective cohort | 1 | 2 | 477 children, inclusion criteria for age 7-18 years, mean age of children 13.5 years, cancer. | 2 PROMs.  Pediatric Patient-Reported Outcomes version of the Common Terminology Criteria for **Adverse Events** (Ped-PRO-CTCAE) assessing patient-reported AEs during treatment determining the presence, severity, and interference with daily activities of subjective cancer treatment AEs as reported by children 7-18 years. Clinicians select AEs from the Ped-PRO-CTCAE library for inclusion in a clinical trial or for assessing clinical concerns. For this study, patients completed items for the 15 most frequently occurring AEs.  PROMIS Pediatric **Psychological Stress** assessing children’s cognitive, psychological, and somatic states. Generic. |
| (Hoffmann et al., 2021) Germany and Czech | To present the implementation of the MyPal-CHILD platform, emphasizing on the AquaScout serious game per se, also presenting the validation process applied before operating the platform in real-world conditions. | Intervention development study | 1 | 2 | Number of children, parents and healthcare personnel NR, inclusion criteria for age 6-17 years, mean age of children NR, cancer. | 2 PROMs.  **Symptom Screening** in Pediatrics Tool (SSPedi) tailored to mobile app and self-reported through gamification. Generic.  PedsQL (not specified module or version) assessing **HRQL.** |
| (Houwen-van Opstal, Jansen, van Alfen, & de Groot, 2014) Netherlands | To investigate the relationship between HRQL and disease severity in boys with Duchenne muscular dystrophy (DMD), to compare the boys´ HRQL perceptions with that of their parents, and to examine the correlation between HRQL and dimensions of the International Classification of Functioning, Disability and Health model in boys with DMD. | Case-control study | 3 | 2 | 40 children, number of parents NR, inclusion criteria for age 8-20 years, mean age of children 11.5, DMD. | 1 PROM.  KIDSCREEN-52 assesses **HRQL**, generic measure of HRQL in children aged 8 to 18 years, 10 domains, containing self-report forms in addition to the parent report forms. Generic. |
| (Huang, Wen, Revicki, & Shenkman, 2011) USA | To validate psychometric properties of a generic HRQL instrument, the PedsQL 4.0, for children with life-threatening conditions. | Validation study | mixed | 2 | 257 children and 257 parents, inclusion criteria for age 2-18 years, mean age of children 11.4 years, various diagnosis (not specified, only life-threatening). | 1 PROM.  PedsQL Core 4.0 measuring **HRQL**. Generic. |
| (Husson et al., 2017) The Netherlands | To examine changes in HRQL and its predictors during the first 2 years after initial cancer diagnosis in adolescent and young adults (AYAs) patients with cancer. | Prospective cohort study | 1 | 2 | 215 children, inclusion criteria for age 15-39 years, mean age of children 23.6 years, cancer. | 1 PROM.  SF-36 Medical Outcomes Study Short Form-36 Health Survey to assess **HRQL**  in 8 aspects of physical and mental health functioning: physical functioning, role function-physical, bodily pain, social functioning, mental health, role function-emotional, vitality, and general health. Generic. |
| (Ilowite et al., 2018) USA | To test the association between household income and symptom distress and HRQL. To investigate the impact of socioeconomic status on health outcomes in children with cancer. | Cross-sectional study | 1 | 2 | 78 children and 78 parents, inclusion criteria for age 2 years and older, mean age of children NR, cancer. | 2 PROMs  PediQUEST Memorial Symptom Assessment Scale (PQ-MSAS) measuring the frequency, severity, and extent of bother for 24 **symptoms**. PQ-MSAS versions eliciting patient-reported symptom distress. Parent-proxy versions of the PQ-MSAS used for very young children and older children who declined to self-report. Disease-specific.  Pediatric Quality of Life Inventory 4.0 (PedsQL) assessing **HRQL.** Generic. |
| (Jibb et al., 2017) Canada | To evaluate the implementation of the app to inform a future randomized controlled trial (RCT) and obtain treatment effect estimates for pain intensity, pain interference, HRQL, and self-efficacy. | Pilot study | 1 | 2 | 40 children, inclusion criteria for age 12-18 years, mean age of children 14.2 years, cancer. | 3 PROMs  Brief pain inventory (BPI) assessing **pain.** Generic.  PROMIS pediatric pain interference short form scale assessing **pain interference**. Generic.  PedsQL 4.0 assessing self-reported **HRQL**. Generic. |
| (Lafond, Kelly, Hinds, Sill, & Michael, 2015) USA | To evaluate the feasibility and outcomes of early palliative care consultation for children and adolescents with high-risk or advanced cancers and other potentially life-limiting nonmalignant diseases undergoing hematopoietic stem cell transplantation. | Feasibility study | 1 | 2 | 12 children and 12 parents, inclusion criteria for age 0-18 years, mean age of children 8.8 years, cancer. | 2 PROMs  Children’s Comfort Daisies measuring self-reported **comfort** from the child’s perspective (3-7 years) or the 1-question assessment on the Comfort Daisies, a 4-point Likert scale that has been validated with children ages 3 to 7 years. Generic.  Comfort Line Visual Analog scale (7-21 years) measuring **comfort** with ratings from 1 (very comfortable) to 10 (very uncomfortable). Generic. |
| (Lau et al., 2020) USA | To examine whether response to Promoting Resilience in Stress Management differed across key sociodemographic characteristics (sex, age, race, and relative neighborhood socioeconomic disadvantage). | Post-hoc analysis of a randomized controlled trial | 1 | 2 | 92 children, inclusion criteria for age 12-25 years, mean age of children NR, cancer. | 5 PROMs  The Connor Davidson Resilience Scale (CDRISC-10) assessing self-perceptions of **resilience**. Generic.  The Benefit and Burden Scale for Children assessing perceived **benefits and burdens of illness**. Generic.  PedsQL generic assessing **HRQL.** Generic.  PedsQL cancer module assessing **cancer related HRQL.** Disease-specific.  The Kessler 6 Psychological Distress Scale (K-6) assessing global psychological **distress**. Generic. |
| (Leahy et al., 2021) USA | To deploy patient-reported symptom monitoring in hospitalized pediatric patients with cancer for routine clinical care. | Feasibility study | 1 | 2 | 52 children and 52 parents, inclusion criteria for age 7-18 years, mean age of children 11.0 years, cancer. | 1 PROM  Ped-PRO-CTCAE: pediatric Patient-Reported Outcomes version of the Common Terminology Criteria for **Adverse Events.** Disease-specific. |
| (Lee, Boyle, Zaslowe-Dude, Wolfe, & Marcus, 2020) USA | To explore parental perceptions of palliative radiation therapy (pRT). | Prospective cohort study | 1 | 2 | 28 children and 37 parents, inclusion criteria for age NR, mean age of children NR, cancer. | 1 PROM  Study specific, items on palliative course**, side effects** of radiotherapy and outcomes at three months reported by parent proxy. Disease-specific. |
| (Levine et al., 2017) USA | To determine the perception of symptom burden early in treatment and assess attitudes toward early integration of palliative care in pediatric oncology patient-parent pairs,  and to establish whether patients have unmet needs at the initiation of cancer therapy and whether patient and family attitudes are indeed a barrier to early palliative care. | Prospective cohort study | 1 | 2 | 129 children and 129 parents, inclusion criteria for age 10-18 years, mean age of children 14.0 years, cancer. | 1 PROM  Study specific assessing **symptom burden and suffering** (a novel survey instrument, one patient survey and one parent survey developed in a 9-part stepwise process.) Disease-specific. |
| (Limbers et al., 2011) USA | To compare the level of HRQL of pediatric liver transplant recipients to children with other chronic health conditions that  share similar non-categorical characteristics with  LT recipients. | cross-sectional study | mixed | 2 | 873 children and 869 parents, inclusion criteria for age 2-18 years, mean age 8.17 years, various diagnosis (liver TX compared with JRA, type 1 diabetes, cancer in remission, cardiac disease, end-stage renal disease, and inflammatory bowel disease). | 1 PROM  PedsQL 4.0 generic core scale measuring **HRQL**. Generic. |
| (Lykke, Ekholm, Olsen, & Sjogren, 2021) Denmark | To investigate the prevalence and intensity of symptoms and problems in children ≥3 years with life-limiting diagnoses during the last month of life assessed by their parents. | Retrospective cross-sectional survey | mixed | 3 | 152 deceased children and 193 parents, inclusion criteria for age 0-18, mean age of children NR as the children are deceased, various diagnosis. | 1 PROM  “To lose a child” - modified version adapted to the sample and language assessing symptoms and problems affecting **well-being** the last month of life. Generic. |
| (Lyon et al., 2008) USA | To examine the frequency of do-not-resuscitate (DNR) orders and hospice enrollment in children/adolescents living with acquired immune deficiency syndrome (AIDS) and followed in Pediatric AIDS Clinical Trials Group (PACTG) Study 219c, and evaluated for any association with racial disparities or enhanced QOL, particularly psychological adjustment. | Cross-sectional study | 2 | 2 | 726 children and number of parents NR, inclusion criteria for age NR, mean age of children 12.9 years, HIV/AIDS. | 1 PROM  General Health Assessment for Children (GHAC) measuring six **QOL** domains: general health perception; symptom distress; physical functioning; psychological functioning; social/role functioning; and health care utilization. Tailored to a pediatric population. Generic. |
| (Madden et al., 2019) USA | To determine the feasibility of using a set of questions that assessed how bothersome common physical and psychological symptoms were in children with cancer referred to a pediatric palliative care service. | Prospective cohort study | 1 | 2 | 83 children and 107 parents, inclusion criteria for age 7-18 years, mean age of children 12.7 years, cancer. | 1 PROM  Study specific symptom assessment questions based on ESAS, MSAS and PQ-MSAS. The symptom assessment questions assess 11 physical and psychological **symptoms**. Disease-specific. |
| (Mandrell et al., 2016) USA | To assess HRQL from the time of diagnosis until disease progression in a cohort of children with diffuse intrinsic pontine glioma (DIPG). | Prospective cohort study | 1 | 1 | 25 children and 25 parents, inclusion criteria for age 2-17 years, mean age of children NR, cancer. | 2 PROMs  PedsQL 4.0. This instrument has parallel forms for child and parent report, with higher scores reflecting a higher **HRQL**. Generic.  PedsQL 3.0 Brain Tumor Scale measuring **HRQL** in children and adolescents who are receiving treatment or are survivors of pediatric brain tumors. The module contains six scales and addresses cognitive function, pain and hurt, movement and balance, procedural anxiety, nausea, and worry. This instrument has parallel forms for child and parent report. Disease-specific. |
| (Mellion et al., 2014) USA | To compare HRQL in a group of pediatric patients with CHD and healthy controls and patients with other chronic diseases, and to compare HRQL among patients with CHD of various severity categories with one another, with controls, and with patients with other chronic diseases. | Cross-sectional study | mixed | 2 | 1138 children and 1138 parents, inclusion criteria for age 8-18 years, mean age NR, various diagnosis (CHD compared to healthy controls and other chronic diseases). | 1 PROM  PedsQL 4.0 Generic Core Scales scores (aged 8-12 years) and adolescents (aged 13-18 years) assessing **HRQL.** Generic. |
| (Meryk et al., 2021) Austria | To evaluate the feasibility and value of daily patient-reported outcome measures (PROMs) by children receiving chemotherapy for cancer. | Feasibility study | 1 | 2 | 12 children and number of parents NR, inclusion criteria for age 5-18 years, mean age of children 7.2 years, cancer. | 1 PROM  ePROtect - **symptoms burden** assessing symptom burden on and after treatment. Disease-specific. |
| (Montgomery et al., 2020) USA | To evaluate the feasibility of electronic data collection in children with advanced cancer using self-report of symptom frequency, severity, and distress. | Feasibility study | 1 | 2 | 46 children, inclusion criteria for age 7-18 years, mean age 13.0 years, cancer. | 1 PROM  PQ-MSAS 7-12 (8 items) and abbreviated PQ-MSAS 13-18 (11 items). PQ-MSAS 7-12: **Illness symptom** (pain, fatigue, sadness, worry, appetite, nausea, sleeping difficulties, shortness of breath), frequency, severity, and distress. PQ-MSAS 13-18: same as above and additionally: diarrhea, constipation, irritability. Disease-specific. |
| (Morley et al., 2014) Canada | To describe the third phase of the PAC-QOL instrument development. | Outcome development study | 1 | 2 | 34 children and 40 parents, inclusion criteria for age 2-18 years, mean age 12.61 years, cancer. | 1 PROM  Pediatric Advanced Care-**Quality of Life** Scale (PAC-QOL) including Physical Comfort, Psychological Well-Being, Social Interaction, Resilience, and Quality of Care. Four report versions: two self-reports (child self-report for ages 8-12; adolescent self-report for ages 13-18) and two parent reports (parent report for toddlers ages 2-4; and parent report for children and adolescents ages 5-18). Generic. |
| (Namisango et al., 2022) Kenya, Uganda, South Africa | To determine the face and content validity, comprehensiveness, comprehensibility, acceptability, and feasibility, and implementability of the C-POS. | Mixed methods | mixed | 2 | 6 children and 16 parents, inclusion criteria for age 7-17 years, mean age NR, diagnosed with life limiting and life-threatening illness. | 1 PROM  Children's palliative outcome scale (C-POS) assesses **palliative outcomes**. A short 12-item instrument combining self-report (child) and proxy (parents) reports regarding health status, HRQL, and QOL. It covers four dimensions: physical and psychological symptoms, information received, advance care planning, and self-efficacy. Generic. |
| (Neul, Minard, Currier, & Goldstein, 2013) USA | To report the first data on longitudinal change in global (PedsQLTM4.0) and disease specific (PedsQLTM3.0 ESRD Module) HRQL pediatric ESRD patient and proxy ratings. | Prospective cohort | 1 | 2 | 53 children and number of parents NR, inclusion criteria for age 2-17 years, mean age of children 12.1 years, ESRD. | 2 PROMs  PedsQL 3.0 ESRD Module to assess **HRQL** in children 2-18. Disease-specific.  PedsQL 4.0 **HRQL** in children 2-18. Generic. |
| (Obiagwu, Sangweni, Moonsamy, Khumalo, & Levy, 2018) South Africa | To analyze the HRQL of children and adolescents with end-stage renal disease on dialysis in Johannesburg. | Cross-sectional study | 1 | 2 | 27 children and number of parents NR, inclusion criteria for age 5-25 years, mean age of children 14.4 years, ESRD. | 1 PROM  PedsQL 3.0 ESRD Module **HRQL** in children 2-18. Disease-specific. |
| (Park, Hwang, et al., 2012) Korea, A. Same study as Park et al 2012 B. | To assess HRQL in children and adolescents with ESRD using the Korean version of PedsQL ESRD. | Cross-sectional study | 1 | 2 | 92 children and 92 parents, inclusion criteria for age 2-18 years, mean age of children 12.1 years, ESRD. | 1 PROM  PedsQL 3.0 ESRD Module **HRQL** in children 2-18. Disease-specific. |
| (Park, Cho, et al., 2012) Korea, B. Same study as Park et al 2012 A | To assess the psychometric properties of the Korean translation of the PedsQL End Stage Renal Disease module. | Validation study | 1 | 2 | 92 children and 92 parents, inclusion criteria for age 2-18 years, mean age of children 12.1 years ESRD. | 2 PROMs  PedsQL 3.0 ESRD Module **HRQL** children 2-18. Disease-specific.  PedsQL 4.0 Generic module **HRQL** in children 2-18. Generic. |
| (Parsons, Fairclough, Wang, & Hinds, 2012) USA | To explore the relationship between child self-report and parent proxy report of HRQL by domain, using the Pediatric Quality of Life Inventory (PedsQL) 4.0 Generic Core Scales in a cohort of children newly diagnosed with cancer. | Prospective cohort | 1 | 1 | 222 children and 222 parents, inclusion criteria for age 5-18 years, mean age of children 11.6 years, cancer. | 1 PROM  PedsQL 4.0 Generic module **HRQL** in children 2-18. Generic. |
| (Phipps et al., 2012) USA | To evaluate the efficacy of complementary health-promotion interventions designed to reduce distress and promote well-being for children undergoing stem cell transplantation interventions. | Randomized controlled trial | 1 | 2 | 171 children and 171 parents, inclusion criteria for age 6-18 years, mean age of children 12.8 years, cancer. | 4 PROMs  Children’s Depression Inventory (CDI) is a 27-item self-report measure assessing **depressive** symptomatology developed for kids 7-17. Generic.  Posttraumatic Stress Disorder Reaction Index (PTSDI) is a 22-item measure used to assess **symptoms of posttraumatic stress disorder**/posttraumatic stress syndrome (PTSD/PTSS) in children who have experienced a traumatic event. Both child self-report and parent-proxy. Generic.  Children’s Health Questionnaire measures **HRQL** for children, both child self-report and parent proxy. Generic.  **Benefit** finding scale for children (BFSC). Generic. |
| (Pritchard et al., 2008) USA | To identify the cancer-related symptoms that most concerned parents during the last days of their child’s life and the strategies parents identified as helpful with their child’s care. | Retrospective survey | 1 | 3 | 52 deceased children and 65 parents, inclusion criteria for age 0-21 years, mean age NR as the children were deceased, cancer. | 1 PROM  Study specific, 3 primary **symptoms** questions to identify primary symptoms at last days/ death through parent proxy report. |
| (Rensen et al., 2020) Netherland | To compare paternal and maternal proxy reports, and explore determinants of couple disagreement (sociodemographic and medical characteristics, and parental QOL and distress). | Cross sectional study | 1 | 2 | 120 children and 240 parents, inclusion criteria for age 2 years and up, mean age of children 11.0 years, cancer. | 1 PROM  PedsQL 4.0 core scales proxy version (2-18) years, Dutch version, assessing **HRQL** in children as assessed by parent proxy. Generic. |
| (Requena et al., 2022) USA | To identify barriers to effective symptom management in pediatric advanced cancer. | Qualitative multiple case study | 1 | 2 | 23 children and 23 parents, inclusion criteria for age 2 years and up, median age of children 13.0 years, cancer. | 2 PROMs  PQ-MSAS assessing **symptoms** in cancer in children. Disease-specific.  PedsQL 4.0 generic core assessing **HRQL.** Generic. |
| (Roizen et al., 2008) Argentina | To validate the Argentinean Spanish version of the PedsQL 4.0 Generic Core Scales in Argentinean children and adolescents with chronic conditions and to assess the impact of socio-demographic characteristics on the instrument's comprehensibility and acceptability. | Validation study | Mixed | 2 | 287 children and 287 parents, inclusion criteria for age 2-18 years, mean age of children NR, various diagnosis: Allogeneic Hematopoietic Stem Cell Transplantation (SCT), COPD, HIV/AIDS, Cancer, ESRD requiring dialysis or transplant, or a Complex Congenital Cardiopathy (CCC). | 2 PROMs  PedsQL 4.0 generic core assessing **HRQL**  Study specific **overall health**. Children 5 years old and above and their proxies were asked to independently score how they considered the child was feeling over the last month. Generic. |
| (Rosenberg et al., 2016) USA | To describe patient reported HRQL and its relationship to symptom distress. | Prospective, longitudinal study | 1 | 2 | 104 children and number of parents NR (16% of the responses), inclusion criteria for age 2 years and up, mean age of children NR, cancer. | 2 PROMs  PediQuest (PQ) with PedsQL 4.0 and PQ-MSAS.  PedsQL 4.0 measuring **HRQL**, Both self-report and proxy versions. Generic.  PQ-MSAS measuring frequency, severity, and extent of bother from 24 physical and psychological **symptoms**. Three age-appropriate versions (2-6, 7-12, 13-18 years old). Disease-specific. |
| (Rosenberg et al., 2018) USA | To explore the predictive value of screening for distress alone, hope alone, or a combination of both. | Prospective cohort | 1 | 2 | 37 children and 40 parents, inclusion criteria for age 14-25 years, mean age of children NR, cancer. | 1 PROM  PedsQL 3.0 Cancer Module assessing **HRQL**. Disease-specific. |
| (Salaverria et al., 2021) El Salvador | To determine if curative intent at relapse or induction failure, when compared with palliative intent, was associated with child’s physical health, pain or general fatigue and parents’ QOL over time among patients with pediatric leukemia in El Salvador. | Prospective cohort study | 1 | 2 | 60 children and 60 parents, inclusion criteria for age 2-18 years, mean age of children NR, cancer. | 3 PROMs  PedsQL 4.0 Generic Core assessing **HRQL.**  PedsQL 3.0 Cancer Module assessing **HRQL.** Disease-specific.  PedsQL Multidimensional Fatigue Scale assessing **fatigue**. Generic. |
| (Schulte et al., 2019) Canada | To assess the feasibility of implementing a tool, or set of tools, capable of screening for psycho-social distress in pediatric cancer patients across the cancer continuum (on treatment, off treatment). | Feasibility study | 1 | 2 | 95 children and 95 parents, inclusion criteria for age 8-18 years, mean age of children 11.5 years, cancer. | 2 PROMs  Distress thermometer, measures **emotional distress** in both self-report and proxy. Generic.  PedsQL Generic Core 4.0, measures **HRQL** Generic. |
| (Schwartz & Brumley, 2017) USA | To examine health-related hindrance (HRH) of personal goals among adolescents receiving treatment for cancer and healthy peers. | Cross sectional study | 1 | 2 | 102 children and number of parents NR, inclusion criteria for age 13-19 years, mean age of children 16.7 years, cancer. | 4 PROMs  PedsQL cancer assessing **HRQL.** Disease-specific.  PedsQL **fatigue.** Generic.  Study specific. Perceived **life threat** was assessed using an item adapted from the Assessment of Life Threat and Treatment Intensity Questionnaire for adolescents ‘‘I could die from my cancer.’’ Three items of the Varni/Thompson Pediatric Pain Questionnaire assessed current pain severity, and frequency and severity of pain in the past 4 weeks. Disease-specific.  Child Depression Inventory CDI, **Depressive symptoms** last 2 weeks. Generic. |
| (Selewski et al., 2014) USA | To evaluate the sensitivity of the PROMIS instruments to pediatric CKD severity (Stage I-III, Stage IV-V, and Renal Transplant), and activity indicators, such as edema and recent hospitalizations. | Cross-sectional study | 1 | 2 | 233 children, inclusion criteria for age 8-17 years, mean age of children NR, ESRD. | 1 PROM with multiple foci  PROMIS PROFILE. Domains related to **depression, anxiety, social-peer relationships, pain interference, fatigue, mobility, and upper extremity function**. Generic. |
| (Shultz et al., 2017) USA | To compare retrospective parent report and electronic medical record documentation of symptoms and to examine associations with parent perceptions of infants suffering at EOL. | Retrospective study | mixed | 3 | 40 deceased children, 67 parents, and number of healthcare personnel NR, inclusion criteria infants, mean age NR as the sample was deceased, various diagnosis (at birth they had respiratory, congenital, heart, prematurity, neurologic, genetic, other). | 2 per child, 3 in the study due to age specific PROMs  Study specific on **symptoms and suffering**, wide range, respiratory distress, pain, agitation, lethargy. Parent proxy report. Disease-specific.  Face, legs, activity, cry, and consolability (FLACC) HCP to assess **pain**, applied by proxy HCP or parent. Generic.  N-PASS by HCP to assess **pain** by HCP proxy. Generic. |
| (Splinter et al., 2018) Netherlands, Belgium, Germany | To assess HRQL across three renal replacement therapy modalities (preemptive transplant, non-preemptive transplant, and dialysis) in comparison with the healthy norm and other chronic health conditions, and to explore related patient factors. | Cross-sectional study | 1 | 2 | 192 children, inclusion criteria for age 8-18 years, mean age of children 13.6 years, ESRD. | 1 PROM  PedsQL, Pediatric Quality of Life Inventory 4.0 assessing **HRQL**. Generic. |
| (Stenmarker, Mellgren, Matus, Schroder Hakansson, & Stenmarker, 2018) Argentina, Sweden | To explore HRQL in children with cancer in two countries, Argentina and Sweden, which have different cultural contexts Specific aims were: to determine HRQL by gender, age, diagnosis, treatment modality, time since diagnosis, and parental education/employment across cultures. Further aims were to assess the child/parent relationship in HRQL and the influence of demographic variables in psychosocial and physical HRQL in each country. | Cross sectional study | 1 | 2 | 58 children and 62 parents, inclusion criteria for age 2-18 years, mean age of children 8.7 years, cancer. | 3 PROMs  PedsQL 4.0 generic to assess **HRQL.** Generic.  PedsQL cancer assess **HRQL.** Disease-specific.  PedsQL fatigue assess **HRQL.** Generic. |
| (Szepetowski et al., 2021) France | To describe the feasibility of using SSPedi administration among childhood and adolescents’ cancer patients with high-risk malignancies. | Feasibility study | 1 | 2 | 15 children, inclusion criteria for age 8-18 years, mean age of children 13.0 years, cancer. | 1 PROM  Symptom Screening in Pediatrics Tool (SSPedi) measure the extent of bothersome **symptoms** among children between 8 and 18 years of age. Generic. |
| (Tanasansuttiporn et al., 2022) Thailand | To identify predictors of HRQL in children who received the modified Blalock–Taussig shunt and those who underwent total repair. | Historic cohort | 1 | 2 | 148 children and 232 parents, inclusion criteria for age 1-18 years, mean age of children NR, cyanotic heart disease. | 1 PROM  PedsQL 4.0 generic core, in Thai assess **HRQL.** Generic. |
| (Thrane, Williams, Grossoehme, & Friebert, 2022) USA | To evaluate the effects of Reiki on pain, stress, heart, and respiratory rates, oxygenation, QOL in hospitalized young children receiving palliative care services. | Pilot study | mixed | 2 | 16 children and 16 parents, number of healthcare personnel NR, inclusion criteria for age of children 1-5 years, mean age 2.2 years, various, cancer, congenital, genetic, other. | 2 PROMs  Child health status one item. Generic  PedsQL 4 generic core to assess **HRQL.** Generic. |
| (Tiwari et al., 2015) India | To assess the QOL of children with ESRD on maintenance dialysis and following kidney transplant and to compare the HRQL assessed by child self-report and parent’s proxy report. | Cross-sectional study | 1 | 2 | 55 children and 55 parents, inclusion criteria for age 2-18 years, mean age of children NR, kidney TX and ESRD. | 1 PROM  PedsQL 3.0 ESRD module, Hindi version, assess **HRQL** in children with ESRD. Disease-specific. |
| (Tomlinson, Hinds, Bartels, Hendershot, & Sung, 2011) A  Canada | To compare QOL of children with cancer with no reasonable chance of cure reported by parents 6 months or fewer versus more than 6 months before death. | Cross-sectional study | 1 | 2 | 73 children and 73 parents, inclusion criteria for age 2-18 years, mean age of children NR, cancer. | 3 PROMs  PedsQL 4.0 Generic Core Scales to assess **HRQL.** Generic.  PedsQL Acute Cancer Module to assess **HRQL.** Disease-specific.  PedsQL Multidimensional Fatigue Scale to assess **HRQL.** Generic. |
| (Tomlinson, Hendershot, et al., 2011) B  Canada | To describe concordance between fathers’ and mothers’ evaluation of QOL and determine correlation between mother and father for how factors such as hope, anticipated QOL, and prolonged survival time influence the decision between supportive care alone versus aggressive chemotherapy. | Cross-sectional study | 1 | 2 | 13 children and 26 parents, inclusion criteria for age NR, mean age of children NR, cancer. | 3 PROMs.  PedsQL 4.0 Generic Core Scales assessing **HRQL.** Generic.  PedsQL Acute Cancer Module assessing **HRQL.** Disease-specific.  PedsQL Multidimensional Fatigue Scale assessing **HRQL.** Generic. |
| (Tomlinson et al., 2014) Canada | To identify if any of these scales were suitable for use or adaptation as a self-report symptom screening tool, and if not, to begin the process of creating a new tool. | Development study | 1 | 2 | Development of a new tool for self-report of symptoms in cancer among children 2 years and older, and 11 healthcare personnel participated. | 1 PROM  Symptom Screening in Pediatrics Tool (SSPedi) **symptom** screening. Generic. |
| (Tomlinson et al., 2021) Canada | To finalize the approach to co-SSPedi administration with instruction that is easy to understand, resulting in dyads completing co-SSPedi correctly. | Development study | 1 | 2 | 60 children and 60 parents, number of healthcare personnel NR, inclusion criteria for age 4-18 years, mean age of children NR, cancer. | 1 PROM  Co- Symptom Screening in Pediatrics Tool (Co-SSPedi) self-report **symptom** screening tool. Generic. |
| (Ullrich et al., 2010) USA | To describe fatigue experienced by children with advanced cancer and to identify the factors associated with suffering from fatigue and its treatment. | Retrospective cross-sectional study | 1 | 3 | 141 deceased children and 141 parents, inclusion criteria for age NR, mean age of children NR, cancer. | 1 measure with PRO items  Study specific 390-item semi-structured questionnaire on **symptoms** such as fatigue and associated factors, not all items are PROM. Disease-specific. |
| (Ullrich et al., 2018) USA | To describe fatigue as prospectively reported by children with advanced cancer and to identify the factors associated with fatigue and associated distress. | Pilot randomized controlled trial | 1 | 3 | 104 children and 104 parents, inclusion criteria for age 2 years and up, mean age of children NR, cancer. | 1 PROM  PQ MSAS through PediQUEST on **symptoms** in cancer in children. Disease-specific. |
| (Unay et al., 2020) Turkey | To investigate QOL and the prevalence of anxiety, depression, and post-traumatic stress disorder in children and adolescents before and after liver transplantation and to compare them with healthy controls. | Cross sectional study | 1 | 2 | 50 children, inclusion criteria for age 5-18 years, mean age of children 11.25 years, liver TX. | 3 PROMs  PedsQL to assess **HRQL.** Generic.  SCARED screen for **anxiety related emotional disorders.** Generic  Children depression inventory CDI to assess **symptoms of depression**. Generic. |
| (Vahsen, Broder, Hraska, & Schneider, 2018) Germany | To assess a broad range of neuropsychological outcome variables in children with functionally single ventricle hearts after a total Cavo pulmonary connection and to examine potential risk factors for impaired neurodevelopment. | Cross-sectional study | 1 | 2 | 104 children and number of parents NR, inclusion criteria for age 2-10 years, mean age of children 8.6 years, heart disease. | 1 PROM  Inventory for the assessment of the **QOL** in children and adolescents (ILK). Generic. |
| (Van Cleve, Muñoz, Savedra, et al., 2012) A USA. Same study as Van Cleve et al 2012, B. | To examine pain symptoms and management in children with advanced cancer using child self-report and nurse documentation. | Prospective cohort | 1 | 2 | 62 children and number of healthcare personnel NR, inclusion criteria for age 6-17 years, mean age of children NR, cancer. | 2 PROMs  MSAS to assess 30 **symptoms** across physical, psychological, and global distress as reported by children. Disease-specific.  Body outline to provide pain location. Generic. |
| (Van Cleve, Muñoz, Riggs, Bava, & Savedra, 2012) B USA. Same study as Van Cleve et al 2012, A. | To examine the common symptoms and to explore commonly occurring symptoms over time in children with advanced cancer. | Prospective cohort | 1 | 2 | 60 children and number of healthcare personnel NR, inclusion criteria for age 6-17 years, mean age of children NR, cancer. | 1 PROM  MSAS to assess 30 **symptoms** across physical, psychological, and global distress as reported by children. Disease-specific. |
| (Varni, Limbers, & Burwinkle, 2007) USA | To compare generic HRQL across ten chronic disease clusters and 33 disease categories/severities from the perspectives of patients and parents. Comparisons were also bench marked with healthy children’s data. | Cross-sectional | mixed | 2 | 2500 children and number of parents NR, inclusion criteria for age 2-18 years, mean age of children 12.0 years, various diagnosis (diabetes, gastrointestinal conditions, cardiac conditions, asthma, obesity, end stage renal disease, psychiatric disorders, cancer, rheumatologic conditions, and cerebral palsy). | 1 PROM  PedsQL 4.0 to assess **HRQL**. Generic. |
| (Vollenbroich et al., 2016) Switzerland | To analyze symptom perception by parents and healthcare professionals and the quality of symptom management in a pediatric palliative home care setting and identify which factors contribute to a high quality of palliative and EOL care for children. | Retrospective, cross-sectional study | mixed | 3 | 38 deceased children and 38 bereaved parents, inclusion criteria for age NR, mean age of children NR as the sample was deceased, various diagnosis (congenital, oncological, neurological, cardiac, and other disorders). | 1 PROM  Study specific on **symptom** perception and treatment, 56 items with a list up to four major symptoms and assess severity on a Likert scale. Generic. |
| (Vrijmoet-Wiersma et al., 2009) the Netherlands | To assess the (1) self- and proxy-reported HRQL compared to a norm group, (2) levels of parenting stress compared to a norm group, (3) differences in HRQL and parenting stress pre- and post-SCT, and (4) effect of child age and parenting stress on self- and proxy-reported HRQL pre- and post-SCT. | Pre-poststudy | 1 | 2 | 21 children and 31 parents, inclusion criteria for age 3 years and up, mean age of children 11.0 years, Malignant Acute myeloid leukemia (AML) and Acute lymphocytic leukemia (ALL) and non-malignant (blood disease, immune disease) diseases. | 1 PROM  DUX 25 Dutch children AZL TNO **QOL** (TACQOL) questionnaire. Generic. |
| (Weaver et al., 2017) USA | To evaluate QOL for pediatric patients receiving palliative care consultations and to compare patient-reported QOL with parent perception of the child’s QOL across wellness domains. | Cross-sectional study | mixed | 2 | 10 children and 10 parents, inclusion criteria for age 5-18 years, mean age of children 12.4 years, with various diagnosis, 4 neurodegenerative, 3 cardiac, 2 pulmonary, 1 genetic. | 1 PROM  PedsQL 4.0 assessing dimensions of **HRQL.** Generic. |
| (Weaver et al., 2018) USA | To longitudinally evaluate the effect of time on QOL and family impact for pediatric palliative care patients across all diagnoses and ages. | Prospective cohort | mixed | 2 | 87 children and number of parents NR, inclusion criteria for age 0-16 years, mean age of children 5.1 years, with various diagnosis (neurologic, cardiac, musculoskeletal, pulmonary, and oncologic). | 1 PROM  PedsQL assessing **HRQL**. Generic. |
| (Weaver, Hanna, et al., 2020) A USA | To report on the QOL and family experience for children with spinal muscular atrophy with attentiveness to patient- and proxy-concordance and to stratify quality of life reports by spinal muscular atrophy type and medical interventions. | Prospective cohort | 3 | 2 | 58 children and 58 parents, inclusion criteria for age 0-20 years, mean age of children 8.9 years, with Spinal Muscular Atrophy (SMA) (SMA type 1= 26, SMA type 2=23, SMA type 3= 9). | 1 PROM  PedsQL 3.0 Neuromuscular Module to assess **HRQL** over 3 dimensions: (1) About My Neuromuscular Disease (17 items with emphasis on physical functioning), (2) Communication (3items), and (3) About Our Family Resources (5 items). Self-report and proxy. Disease-specific. |
| (Weaver, Robinson, & Wichman, 2020) B USA | To measure the impact of aromatherapy using validated child-reported nausea, pain, and mood scales 5 minutes and 60 minutes after aromatherapy exposure. | Pilot study | mixed | 2 | 180 children, inclusion criteria for age 4-17 years, mean age of children 9.4 years, with various diagnosis (oncological, hematological, cardiac, pulmonary, neurologic, nephrological, urological, endocrine, orthopedic. | 3 PROMs  Baxter Retching Faces (BARF) visual numeric 1-10 scale to assess **nausea severity**, developed for kids. Generic.  Wong-Baker (FACES) visual numeric 1-10 scale to assess **pain**. Developed for self-report from the ages of 3 and older. Generic.  Children’s Anxiety and Pain Scale (CAPS) visual numeric 1-5 scale for mood aimed at separately assessing **anxiety and pain intensity** through self-report developed for children from 4 to 10 years old. Generic. |
| (Weaver, Shostrom, Neumann, Robinson, & Hinds, 2021) USA | To explore physical and emotional symptom burden and family impact assessments for children with terminal cancer receiving home based-hospice care. | Pilot study | 1 | 3 | 11 children and 11 parents, inclusion criteria for age 7-18 years, mean age of children 11.9 years, cancer. | 1 PROM  Memorial **Symptom** Assessment Scale (MSAS) 7-12 or MSAS proxy 7-12. Disease-specific. |
| (Weaver, Wang, Greenzang, McFatrich, & Hinds, 2022) USA | To explore whether the severity of fatigue, pain, and anxiety as self-reported by children with cancer at baseline may serve as predictors of pediatric patient presence in the Low, Medium, or High Suffering profile. | Prospective cohort | 1 | 2 | 436 children, number of parents NR, inclusion criteria for age NR, mean age of children 13.0 years, cancer. | 6 PROMs  PROMIS **anxiety.** Generic.  PROMIS **depression.** Generic.  PROMIS **fatigue.** Generic.  PROMIS **pain.** Generic.  PROMIS **mobility.** Generic.  Ped-pro-CTCAE assessing **adverse events** in pediatric cancer care. |
| (Weekly, Riley, Wichman, Tibbits, & Weaver, 2019) USA | To explore the impact of massage therapy on pediatric palliative care patient’s symptom burden and medication use pattern, to describe the impact of massage therapy on family caregiver distress, and to report on bedside nursing staff perception of massage therapy for children and their families. | Pre–posttest | mixed | 2 | 53 children, number of parents NR, 135 healthcare personnel, inclusion criteria for age NR, mean age of children 3.8 years, various diagnosis (not specified, complex, chronic medical conditions receiving palliative care, mainly nonverbal, critically ill, or neurologically compromised patients). | 4 or 2 PROMs (DTS by all, and FLACC or the other combined)  Distress thermometer (DTS) is a self-report tool that asks patients to rate their **emotional distress** (and caregivers and medical providers, about the patients) on a visual analog scale designed to appear as a thermometer. Generic.  FLACC. Generic.  FACES pictorial scale to assess **pain** through children self-report. Generic.  Nausea Baxter retching faces (BARF) assess **nausea severity** in children. Generic.  Dalhousie Dyspnea Scale to assess **dyspnea.** Generic.  McMurtry Faces **Anxiety** Scale. Generic. |
| (Wiener, Battles, Zadeh, Widemann, & Pao, 2017) USA | To assess the validity, inter-rater reliability, sensitivity/specificity, acceptability, and feasibility of administration of a pediatric distress thermometer (DT) designed to screen for the presence of psychosocial distress in youth with serious medical illnesses. | Feasibility study | mixed | 2 | 281 children and 281 parents, inclusion criteria for age 7-21 years, mean age of children NR, with various diagnosis (cancer, HIV, NF1, PIDs, DNA repair disease, SSD, Li-Fraumeni). | 5 PROMs (not all in combination due to age)  Distress thermometer (DTS) is a self-report tool that asks patients to rate their **emotional distress** (and caregivers and medical providers, about the patients) on a visual analog scale designed to appear as a thermometer. Generic.  Children’s Depression Inventory for self-report or proxy report of **depressive symptoms** in children. Generic.  The Brief Symptom Inventory 18 (BSI-18) to assess **psychological distress** in medically ill patients. Generic.  Wong-Baker FACES Pain Scale to assess **pain.** Generic.  The Childhood Fatigue Scales to assess **fatigue**. Generic. |
| (Williams, Schmideskamp, Ridder, & Williams, 2006) USA | To (a) assess symptoms manifested by children during chemotherapy by parent report on a checklist, (b) describe the responses of parents/caregivers of children with cancer and the care they provide to alleviate the symptoms, and (c) examine the relationship between parent report of symptoms and clinician rating of child’s functional status. | Pilot study | 1 | 2 | 11 children and 11 parents, inclusion criteria for age 2-18 years, mean age of children 10.4 years, cancer. | 1 PROM  The Therapy-Related Symptom Checklists (TRSC) for adults were adjusted to kids in TRSC-C, assessing **symptoms in cancer** treatment, and for children who prefer to self-report, this version enables them to understand the terms used for each symptom and do a self-report. All the TRSC-C symptoms were reported in one or more of the children by parents/caregivers. Disease-specific. |
| (Wolfe et al., 2014) USA. Same study as Wolfe et al 2015 | To determine whether feeding back patient-reported outcomes (PROs) to providers and families of children with advanced cancer improves symptom distress and HRQL. | Pilot study | 1 | 2 | 104 children number of parents and healthcare personnel NR, inclusion criteria for age 2 years and older, mean age of children NR, cancer. | 3 PROMs.  PediQUEST (PQ) age- and respondent-adapted to assess **symptoms and HRQL**, containing:  PQ-MSAS the Memorial Symptom Assessment Scale  PedsQL core 4.0 assessing **HRQL**  Overall Sickness question, one item. Disease-specific. |
| (Wolfe et al., 2015) USA. Same study as Wolfe et al 2014 | To describe symptom distress in 104 children aged 2 years or older with advanced cancer enrolled onto the Pediatric Quality of Life and Evaluation of Symptoms Technology (PediQUEST) Study (multisite clinical trial evaluating an electronic PRO system). | Pilot study | 1 | 2 | 104 children number of parents and healthcare personnel NR, inclusion criteria for age 2 years and older, mean age of children NR, cancer. | 1 PROM  PQ-MSAS, an adapted version of the validated MSAS. Disease-specific. |
|  | | | | | | |

^A)^ Patient groups as defined by TfSL: Group 1: Life-threatening conditions for which curative treatment may be feasible but can fail; Group 2: Conditions in which premature death is inevitable. Treatment may aim to prolong life and allow normal activities; Group 3: Progressive conditions without curative treatment options. Treatment exclusively palliative may extend over many years; Group 4: Irreversible but nonprogressive conditions causing severe disabilities leading to susceptibility to health complications and likelihood of premature death (Widdas, D., McNamara, K., & Edwards, F. (2013). A core care pathway for children with life-limiting and life-threatening conditions: Together for short lives).

^B)^ Care pathways defined by TfSL: Care pathway 1: Time of diagnosis or recognition of LL/LT condition; Care pathway 2: During ongoing care; Care pathway 3: End of life care (Together for short lives. Care pathways for seriously ill babies, children, young people and their families. Accessed May 19th 2023. https://www.togetherforshortlives.org.uk/changing-lives/supporting-care-professionals/care-pathways/) In addition, Care pathway 0: Perinatal care; Care pathway 4: During transition to adulthood, numbered by the authors.

Akard, T. F., Dietrich, M. S., Friedman, D. L., Wray, S., Gerhardt, C. A., Hendricks-Ferguson, V., . . . Gilmer, M. J. (2020). Randomized Clinical Trial of a Legacy Intervention for Quality of Life in Children with Advanced Cancer. *Journal of palliative medicine, 30*, 30. doi:https://dx.doi.org/10.1089/jpm.2020.0139

Alhusaini, O. A., Wayyani, L. A., Dafterdar, H. E., Gamlo, M. M., Alkhayat, Z. A., Alghamdi, A. S., & Safdar, O. Y. (2019). Comparison of quality of life in children undergoing peritoneal dialysis versus hemodialysis. *Saudi Medical Journal, 40*(8), 840-843. doi:https://dx.doi.org/10.15537/smj.2019.8.12747

Andriastuti, M., Halim, P. G., Kusrini, E., & Bangun, M. (2020). Correlation of Pediatric Palliative Screening Scale and Quality of Life in Pediatric Cancer Patients. *Indian Journal of Palliative Care, 26*(3), 338-341. doi:https://dx.doi.org/10.4103/IJPC.IJPC_197_19

Baek, H. S., Park, K. S., Ha, I. S., Kang, H. G., Cheong, H. I., Park, Y. S., . . . Cho, M. H. (2018). Impact of end-stage renal disease in children on their parents. *Nephrology, 23*(8), 764-770. doi:https://dx.doi.org/10.1111/nep.13083

Baughcum, A. E., Fortney, C. A., Winning, A. M., Dunnells, Z. D. O., Humphrey, L. M., & Gerhardt, C. A. (2020). Healthcare Satisfaction and Unmet Needs Among Bereaved Parents in the NICU. *Advances in Neonatal Care, 20*(2), 118-126. doi:https://dx.doi.org/10.1097/ANC.0000000000000677

Behan, L., Leigh, M. W., Dell, S. D., Quittner, A. L., Hogg, C., & Lucas, J. S. (2019). Validation of pediatric health-related quality of life instruments for primary ciliary dyskinesia (QOL-PCD). *Pediatric Pulmonology, 54*(12), 2011-2020. doi:https://dx.doi.org/10.1002/ppul.24507

Boyden, J. Y., Hill, D. L., Nye, R. T., Bona, K., Johnston, E. E., Hinds, P., . . . Ppcrn Share Project, G. (2022). Pediatric Palliative Care Parents' Distress, Financial Difficulty, and Child Symptoms. *Journal of pain and symptom management, 63*(2), 271-282. doi:https://dx.doi.org/10.1016/j.jpainsymman.2021.08.004

Cheng, L., Yuan, C., Wang, J., & Stinson, J. (2022). Pain Reported by Chinese Children during Cancer Treatment: Prevalence, Intensity, Interference, and Management. *Cancer Nursing, 45(2)*, E345-E354. doi:https://dx.doi.org/10.1097/NCC.0000000000000958

Clave, S., Tsimaratos, M., Boucekine, M., Ranchin, B., Salomon, R., Dunand, O., . . . Berbis, J. (2019). Quality of life in adolescents with chronic kidney disease who initiate haemodialysis treatment. *BMC Nephrology, 20*(1), 163. doi:https://dx.doi.org/10.1186/s12882-019-1365-3

De Bruyne, E., Eloot, S., Vande Walle, J., Raes, A., Van Biesen, W., Goubert, L., . . . Van Hoecke, E. (2022). Validity and reliability of the Dutch version of the PedsQL TM 3.0 End Stage Renal Disease Module in children with chronic kidney disease in Belgium. *Pediatric Nephrology, 37*(5), 1087-1096. doi:https://dx.doi.org/10.1007/s00467-021-05224-3

DeCourcey, D. D., Silverman, M., Oladunjoye, A., & Wolfe, J. (2019). Advance Care Planning and Parent-Reported End-of-Life Outcomes in Children, Adolescents, and Young Adults With Complex Chronic Conditions. *Critical Care Medicine, 47*(1), 101-108. doi:https://dx.doi.org/10.1097/CCM.0000000000003472

Derridj, N., Bonnet, D., Calderon, J., Amedro, P., Bertille, N., Lelong, N., . . . Guedj, R. (2022). Quality of Life of Children Born with a Congenital Heart Defect. *Journal of Pediatrics, 244*, 148-153.e145. doi:https://dx.doi.org/10.1016/j.jpeds.2022.01.003

Dobrozsi, S., Yan, K., Hoffmann, R., & Panepinto, J. (2017). Patient-reported health status during pediatric cancer treatment. *Pediatric blood & cancer, 64*(4), 04. doi:https://dx.doi.org/10.1002/pbc.26295

Dotis, J., Pavlaki, A., Printza, N., Stabouli, S., Antoniou, S., Gkogka, C., . . . Papachristou, F. (2016). Quality of life in children with chronic kidney disease. *Pediatric Nephrology, 31*(12), 2309-2316. Retrieved from http://ovidsp.ovid.com/ovidweb.cgi?T=JS&CSC=Y&NEWS=N&PAGE=fulltext&D=med13&AN=27677977

Eijsermans, R. M., Creemers, D. G., Helders, P. J., & Schroder, C. H. (2004). Motor performance, exercise tolerance, and health-related quality of life in children on dialysis. *Pediatric Nephrology, 19*(11), 1262-1266. doi:http://dx.doi.org/10.1007/s00467-004-1583-0

El Shafei, A. M., Soliman Hegazy, I., Fadel, F. I., & Nagy, E. M. (2018). Assessment of Quality of Life among Children with End-Stage Renal Disease: A Cross-Sectional Study. *Journal of Environmental and Public Health, 2018*, 8565498. doi:https://dx.doi.org/10.1155/2018/8565498

Ellis, G. K., Chapman, H., Manda, A., Salima, A., Itimu, S., Banda, G., . . . Westmoreland, K. D. (2021). Pediatric lymphoma patients in Malawi present with poor health-related quality of life at diagnosis and improve throughout treatment and follow-up across all Pediatric PROMIS-25 domains. *Pediatric Blood and Cancer, 68*(10), e29257. doi:https://dx.doi.org/10.1002/pbc.29257

Feudtner, C., Nye, R., Hill, D. L., Hall, M., Hinds, P., Johnston, E. E., . . . Research Project, G. (2021). Polysymptomatology in Pediatric Patients Receiving Palliative Care Based on Parent-Reported Data. *JAMA Network Open, 4*(8), e2119730. doi:https://dx.doi.org/10.1001/jamanetworkopen.2021.19730

Fladeboe, K. M., O'Donnell, M. B., Barton, K. S., Bradford, M. C., Steineck, A., Junkins, C. C., . . . Rosenberg, A. R. (2021). A novel combined resilience and advance care planning intervention for adolescents and young adults with advanced cancer: A feasibility and acceptability cohort study. *Cancer, 127*(23), 4504-4511. doi:https://dx.doi.org/10.1002/cncr.33830

Fortney, C. A., Pratt, M., Dunnells, Z. D. O., Rausch, J. R., Clark, O. E., Baughcum, A. E., & Gerhardt, C. A. (2020). Perceived Infant Well-Being and Self-Reported Distress in Neonatal Nurses. *Nursing Research, 69*(2), 127-132. doi:https://dx.doi.org/10.1097/NNR.0000000000000419

Fortney, C. A., Sealschott, S. D., & Pickler, R. H. (2020). Behavioral Observation of Infants With Life-Threatening or Life-Limiting Illness in the Neonatal Intensive Care Unit. *Nursing Research, 69*(5S Suppl 1), S29-S35. doi:https://dx.doi.org/10.1097/NNR.0000000000000456

Friedel, M., Brichard, B., Boonen, S., Tonon, C., De Terwangne, B., Bellis, D., . . . Aujoulat, I. (2020). Face and Content Validity, Acceptability, and Feasibility of the Adapted Version of the Children's Palliative Outcome Scale: A Qualitative Pilot Study. *Journal of palliative medicine, 15*, 15. doi:https://dx.doi.org/10.1089/jpm.2019.0646

Goldstein, S. L., Graham, N., Warady, B. A., Seikaly, M., McDonald, R., Burwinkle, T. M., . . . Varni, J. W. (2008). Measuring health-related quality of life in children with ESRD: performance of the generic and ESRD-specific instrument of the Pediatric Quality of Life Inventory (PedsQL). *American Journal of Kidney Diseases, 51*(2), 285-297. doi:https://dx.doi.org/10.1053/j.ajkd.2007.09.021

Goldstein, S. L., Rosburg, N. M., Warady, B. A., Seikaly, M., McDonald, R., Limbers, C., & Varni, J. W. (2009). Pediatric end stage renal disease health-related quality of life differs by modality: a PedsQL ESRD analysis. *Pediatric Nephrology, 24*(8), 1553-1560. doi:https://dx.doi.org/10.1007/s00467-009-1174-1

Grossoehme, D. H., Friebert, S., Baker, J. N., Tweddle, M., Needle, J., Chrastek, J., . . . Lyon, M. E. (2020). Association of Religious and Spiritual Factors With Patient-Reported Outcomes of Anxiety, Depressive Symptoms, Fatigue, and Pain Interference Among Adolescents and Young Adults With Cancer. *JAMA Network Open, 3*(6), e206696. doi:https://dx.doi.org/10.1001/jamanetworkopen.2020.6696

Haller, C., Song, W., Cimms, T., Chen, C. Y., Whitley, C. B., Wang, R. Y., . . . Harmatz, P. (2019). Individual heat map assessments demonstrate vestronidase alfa treatment response in a highly heterogeneous mucopolysaccharidosis VII study population. *Jimd Reports, 49*(1), 53-62. doi:https://dx.doi.org/10.1002/jmd2.12043

Hays, R. M., Valentine, J., Haynes, G., Geyer, J. R., Villareale, N., McKinstry, B., . . . Churchill, S. S. (2006). The Seattle pediatric palliative care project: Effects on family satisfaction and health-related quality of life. *Journal of palliative medicine, 9*(3), 716-728. doi:http://dx.doi.org/10.1089/jpm.2006.9.716

Heath, J., Norman, P., Christian, M., & Watson, A. (2017). Measurement of quality of life and attitudes towards illness in children and young people with chronic kidney disease. *Quality of Life Research, 26*(9), 2409-2419. doi:https://dx.doi.org/10.1007/s11136-017-1605-6

Heye, K. N., Knirsch, W., Scheer, I., Beck, I., Wetterling, K., Hahn, A., . . . Landolt, M. A. (2019). Health-related quality of life in pre-school age children with single-ventricle CHD. *Cardiology in the Young, 29*(2), 162-168. doi:https://dx.doi.org/10.1017/S1047951118001993

Hinds, P. S., Weaver, M. S., Withycombe, J. S., Baker, J. N., Jacobs, S. S., Mack, J. W., . . . Wang, J. (2020). Subjective Toxicity Profiles of Children in Treatment for Cancer: A New Guide to Supportive Care? *Journal of pain and symptom management, 20*, 20. doi:https://dx.doi.org/10.1016/j.jpainsymman.2020.10.017

Hoffmann, S., Schraut, R., Kroll, T., Scholz, W., Belova, T., Erhardt, J., . . . Natsiavas, P. (2021). AquaScouts: ePROs Implemented as a Serious Game for Children With Cancer to Support Palliative Care. *Frontiers in Digital Health, 3*, 730948. doi:https://dx.doi.org/10.3389/fdgth.2021.730948

Houwen-van Opstal, S. L., Jansen, M., van Alfen, N., & de Groot, I. J. (2014). Health-related quality of life and its relation to disease severity in boys with Duchenne muscular dystrophy: satisfied boys, worrying parents--a case-control study. *Journal of Child Neurology, 29*(11), 1486-1495. doi:https://dx.doi.org/10.1177/0883073813506490

Huang, I. C., Wen, P. S., Revicki, D. A., & Shenkman, E. A. (2011). Quality of Life Measurement for Children with Life-Threatening Conditions: Limitations and a New Framework. *Child Indicators Research, 4*(1), 145-160. Retrieved from http://ovidsp.ovid.com/ovidweb.cgi?T=JS&CSC=Y&NEWS=N&PAGE=fulltext&D=prem5&AN=21760876

Husson, O., Zebrack, B. J., Block, R., Embry, L., Aguilar, C., Hayes-Lattin, B., & Cole, S. (2017). Health-Related Quality of Life in Adolescent and Young Adult Patients With Cancer: A Longitudinal Study. *Journal of Clinical Oncology, 35*(6), 652-659. doi:https://dx.doi.org/10.1200/JCO.2016.69.7946

Ilowite, M. F., Al-Sayegh, H., Ma, C., Dussel, V., Rosenberg, A. R., Feudtner, C., . . . Bona, K. (2018). The relationship between household income and patient-reported symptom distress and quality of life in children with advanced cancer: A report from the PediQUEST study. *Cancer, 124*(19), 3934-3941. doi:https://dx.doi.org/10.1002/cncr.31668

Jibb, L. A., Stevens, B. J., Nathan, P. C., Seto, E., Cafazzo, J. A., Johnston, D. L., . . . Stinson, J. N. (2017). Implementation and preliminary effectiveness of a real-time pain management smartphone app for adolescents with cancer: A multicenter pilot clinical study. *Pediatric blood & cancer, 64*(10). doi:https://dx.doi.org/10.1002/pbc.26554

Lafond, D. A., Kelly, K. P., Hinds, P. S., Sill, A., & Michael, M. (2015). Establishing Feasibility of Early Palliative Care Consultation in Pediatric Hematopoietic Stem Cell Transplantation. *Journal of Pediatric Oncology Nursing, 32*(5), 265-277. doi:https://dx.doi.org/10.1177/1043454214563411

Lau, N., Bradford, M. C., Steineck, A., Scott, S., Bona, K., Yi-Frazier, J. P., . . . Rosenberg, A. R. (2020). Examining key sociodemographic characteristics of adolescents and young adults with cancer: A post hoc analysis of the Promoting Resilience in Stress Management randomized clinical trial. *Palliative Medicine, 34*(3), 336-348. doi:https://dx.doi.org/10.1177/0269216319886215

Leahy, A. B., Schwartz, L. A., Li, Y., Reeve, B. B., Bekelman, J. E., Aplenc, R., & Basch, E. M. (2021). Electronic symptom monitoring in pediatric patients hospitalized for chemotherapy. *Cancer, 127*(16), 2980-2989. doi:https://dx.doi.org/10.1002/cncr.33617

Lee, B. K., Boyle, P. J., Zaslowe-Dude, C., Wolfe, J., & Marcus, K. J. (2020). Palliative radiotherapy for pediatric patients: Parental perceptions of indication, intent, and outcomes. *Pediatric blood & cancer, 67*(1), e28003. doi:https://dx.doi.org/10.1002/pbc.28003

Levine, D. R., Mandrell, B. N., Sykes, A., Pritchard, M., Gibson, D., Symons, H. J., . . . Baker, J. N. (2017). Patients' and Parents' Needs, Attitudes, and Perceptions About Early Palliative Care Integration in Pediatric Oncology. *JAMA Oncology, 3*(9), 1214-1220. doi:https://dx.doi.org/10.1001/jamaoncol.2017.0368

Limbers, C. A., Neighbors, K., Martz, K., Bucuvalas, J. C., Webb, T., Varni, J. W., . . . Studies of Pediatric Liver Transplantation Functional Outcomes, G. (2011). Health-related quality of life in pediatric liver transplant recipients compared with other chronic disease groups. *Pediatric Transplantation, 15*(3), 245-253. doi:https://dx.doi.org/10.1111/j.1399-3046.2010.01453.x

Lykke, C., Ekholm, O., Olsen, M., & Sjogren, P. (2021). Paediatric end-of-life care - symptoms and problems: parent assessment. *BMJ supportive & palliative care, 11*, 11. doi:https://dx.doi.org/10.1136/bmjspcare-2021-002891

Lyon, M. E., Williams, P. L., Woods, E. R., Hutton, N., Butler, A. M., Sibinga, E., . . . Oleske, J. M. (2008). Do-not-resuscitate orders and/or hospice care, psychological health, and quality of life among children/adolescents with acquired immune deficiency syndrome. *Journal of palliative medicine, 11*(3), 459-469. doi:https://dx.doi.org/10.1089/jpm.2007.0148

Madden, K., Magno Charone, M., Mills, S., Dibaj, S., Williams, J. L., Liu, D., & Bruera, E. (2019). Systematic Symptom Reporting by Pediatric Palliative Care Patients with Cancer: A Preliminary Report. *Journal of palliative medicine, 22*(8), 894-901. doi:https://dx.doi.org/10.1089/jpm.2018.0545

Mandrell, B. N., Baker, J., Levine, D., Gattuso, J., West, N., Sykes, A., . . . Broniscer, A. (2016). Children with minimal chance for cure: parent proxy of the child's health-related quality of life and the effect on parental physical and mental health during treatment. *Journal of Neuro-Oncology, 129*(2), 373-381. doi:https://dx.doi.org/10.1007/s11060-016-2187-9

Mellion, K., Uzark, K., Cassedy, A., Drotar, D., Wernovsky, G., Newburger, J. W., . . . Pediatric Cardiac Quality of Life Inventory Testing Study, C. (2014). Health-related quality of life outcomes in children and adolescents with congenital heart disease. *Journal of Pediatrics, 164*(4), 781-788.e781. doi:https://dx.doi.org/10.1016/j.jpeds.2013.11.066

Meryk, A., Kropshofer, G., Hetzer, B., Riedl, D., Lehmann, J., Rumpold, G., . . . Crazzolara, R. (2021). Implementation of daily patient-reported outcome measurements to support children with cancer. *Pediatric blood & cancer, 68*(11), e29279. doi:https://dx.doi.org/10.1002/pbc.29279

Montgomery, K. E., Raybin, J. L., Ward, J., Balian, C., Gilger, E., Murray, P., & Li, Z. (2020). Using Patient-Reported Outcomes to Measure Symptoms in Children with Advanced Cancer. *Cancer Nursing, 43*(4), 281-289. doi:http://dx.doi.org/10.1097/NCC.0000000000000721

Morley, T. E., Cataudella, D., Fernandez, C. V., Sung, L., Johnston, D. L., Nesin, A., & Zelcer, S. (2014). Development of the Pediatric Advanced Care Quality of Life Scale (PAC-QoL): evaluating comprehension of items and response options. *Pediatric blood & cancer, 61*(10), 1835-1839. doi:https://dx.doi.org/10.1002/pbc.25111

Namisango, E., Bristowe, K., Murtagh, F. E., Downing, J., Powell, R. A., Atieno, M., . . . Harding, R. (2022). Face and content validity, acceptability, feasibility, and implementability of a novel outcome measure for children with life-limiting or life-threatening illness in three sub-Saharan African countries. *Palliative Medicine*, 2692163221099583. doi:https://dx.doi.org/10.1177/02692163221099583

Neul, S. K., Minard, C. G., Currier, H., & Goldstein, S. L. (2013). Health-related quality of life functioning over a 2-year period in children with end-stage renal disease. *Pediatric Nephrology, 28*(2), 285-293. doi:https://dx.doi.org/10.1007/s00467-012-2313-7

Obiagwu, P. N., Sangweni, B., Moonsamy, G., Khumalo, T., & Levy, C. (2018). Health-related quality of life in children and adolescents with end-stage renal disease receiving dialysis in Johannesburg. *SAJCH South African Journal of Child Health, 12*(2), 58-62. doi:http://dx.doi.org/10.7196/SAJCH.2018.V12I2.1457

Park, K. S., Cho, M. H., Ha, I. S., Kang, H. G., Cheong, H. I., Park, Y. S., . . . Cho, H. Y. (2012). Validity and reliability of the Korean version of the pediatric quality of life ESRD module. *Health & Quality of Life Outcomes, 10*, 59. doi:https://dx.doi.org/10.1186/1477-7525-10-59

Park, K. S., Hwang, Y. J., Cho, M. H., Ko, C. W., Ha, I. S., Kang, H. G., . . . Cho, H. Y. (2012). Quality of life in children with end-stage renal disease based on a PedsQL ESRD module. *Pediatric Nephrology, 27*(12), 2293-2300. doi:https://dx.doi.org/10.1007/s00467-012-2262-1

Parsons, S. K., Fairclough, D. L., Wang, J., & Hinds, P. S. (2012). Comparing longitudinal assessments of quality of life by patient and parent in newly diagnosed children with cancer: the value of both raters' perspectives. *Quality of Life Research, 21*(5), 915-923. doi:https://dx.doi.org/10.1007/s11136-011-9986-4

Phipps, S., Peasant, C., Barrera, M., Alderfer, M. A., Huang, Q., & Vannatta, K. (2012). Resilience in children undergoing stem cell transplantation: results of a complementary intervention trial. *Pediatrics, 129*(3), e762-770. doi:https://dx.doi.org/10.1542/peds.2011-1816

Pritchard, M., Burghen, E., Srivastava, D. K., Okuma, J., Anderson, L., Powell, B., . . . Hinds, P. S. (2008). Cancer-related symptoms most concerning to parents during the last week and last day of their child's life. *Pediatrics, 121*(5), e1301-e1309. doi:http://dx.doi.org/10.1542/peds.2007-2681

Rensen, N., Steur, L. M. H., Schepers, S. A., Merks, J. H. M., Moll, A. C., Kaspers, G. J. L., . . . Grootenhuis, M. A. (2020). Determinants of health-related quality of life proxy rating disagreement between caregivers of children with cancer. *Quality of Life Research, 29*(4), 901-912. doi:http://dx.doi.org/10.1007/s11136-019-02365-9

Requena, M. L., Avery, M., Feraco, A. M., Uzal, L. G., Wolfe, J., & Dussel, V. (2022). Normalization of Symptoms in Advanced Child Cancer: The PediQUEST-Response Case Study. *Journal of pain and symptom management, 63*(4), 548-562. doi:https://dx.doi.org/10.1016/j.jpainsymman.2021.12.009

Roizen, M., Rodriguez, S., Bauer, G., Medin, G., Bevilacqua, S., Varni, J. W., & Dussel, V. (2008). Initial validation of the Argentinean Spanish version of the PedsQL 4.0 Generic Core Scales in children and adolescents with chronic diseases: acceptability and comprehensibility in low-income settings. *Health & Quality of Life Outcomes, 6*, 59. doi:https://dx.doi.org/10.1186/1477-7525-6-59

Rosenberg, A., Orellana, L., Ullrich, C., Kang, T., Geyer, J., Feudtner, C., . . . Wolfe, J. (2016). Quality of life in children with advanced cancer: A report from the pediQUEST study. *Journal of pain and symptom management, 52*(2), 243-253. doi:http://dx.doi.org/10.1016/j.jpainsymman.2016.04.002

Rosenberg, A. R., Bradford, M. C., Bona, K., Shaffer, M. L., Wolfe, J., Baker, K. S., . . . Yi-Frazier, J. (2018). Hope, distress, and later quality of life among adolescent and young adults with cancer. *Journal of Psychosocial Oncology, 36*(2), 137-144. doi:https://dx.doi.org/10.1080/07347332.2017.1382646

Salaverria, C., Plenert, E., Vasquez, R., Fuentes-Alabi, S., Tomlinson, G. A., & Sung, L. (2021). Paediatric relapsed acute leukaemia: curative intent chemotherapy improves quality of life. *BMJ supportive & palliative care, 17*, 17. doi:https://dx.doi.org/10.1136/bmjspcare-2020-002722

Schulte, F., Russell, K. B., Pelletier, W., Scott-Lane, L., Guilcher, G. M. T., Strother, D., & Dewey, D. (2019). Screening for psychosocial distress in pediatric cancer patients: An examination of feasibility in a single institution. *Pediatric Hematology and Oncology, 36*(3), 125-137. doi:https://dx.doi.org/10.1080/08880018.2019.1600082

Schwartz, L. A., & Brumley, L. D. (2017). What a Pain: The Impact of Physical Symptoms and Health Management on Pursuit of Personal Goals Among Adolescents with Cancer. *Journal of Adolescent & Young Adult Oncology, 6*(1), 142-149. doi:https://dx.doi.org/10.1089/jayao.2016.0031

Selewski, D. T., Massengill, S. F., Troost, J. P., Wickman, L., Messer, K. L., Herreshoff, E., . . . Gipson, D. S. (2014). Gaining the Patient Reported Outcomes Measurement Information System (PROMIS) perspective in chronic kidney disease: a Midwest Pediatric Nephrology Consortium study. *Pediatric Nephrology, 29*(12), 2347-2356. doi:http://dx.doi.org/10.1007/s00467-014-2858-8

Shultz, E. L., Switala, M., Winning, A. M., Keim, M. C., Baughcum, A. E., Gerhardt, C. A., & Fortney, C. A. (2017). Multiple Perspectives of Symptoms and Suffering at End of Life in the NICU. *Advances in Neonatal Care, 17*(3), 175-183. doi:https://dx.doi.org/10.1097/ANC.0000000000000385

Splinter, A., Tjaden, L. A., Haverman, L., Adams, B., Collard, L., Cransberg, K., . . . Groothoff, J. W. (2018). Children on dialysis as well as renal transplanted children report severely impaired health-related quality of life. *Quality of Life Research, 27*(6), 1445-1454. doi:http://dx.doi.org/10.1007/s11136-018-1789-4

Stenmarker, E., Mellgren, K., Matus, M., Schroder Hakansson, A., & Stenmarker, M. (2018). Health-related quality of life, culture and communication: a comparative study in children with cancer in Argentina and Sweden. *Journal of Patientreported Outcomes, 2*(1), 49. doi:https://dx.doi.org/10.1186/s41687-018-0075-0

Szepetowski, S., Saultier, P., Andre, N., Pauly, V., Dupuis, L. L., Sung, L., & Revon-Riviere, G. (2021). Symptom Screening in Pediatrics Tool in children and adolescents with high-risk malignancies: a pilot study. *BMJ supportive & palliative care, 15*, 15. doi:https://dx.doi.org/10.1136/bmjspcare-2020-002753

Tanasansuttiporn, J., Oofuvong, M., Wasinwong, W., Chittithavorn, V., Duangpakdee, P., Jarutach, J., & Yunuswangsa, Q. (2022). Predictors of Health-Related Quality of Life in Children with Cyanotic Heart Disease Who Underwent Palliative and Total Repair. *Congenital Heart Disease, 17(3)*, 245-267. doi:https://dx.doi.org/10.32604/chd.2022.021778

Thrane, S. E., Williams, E., Grossoehme, D. H., & Friebert, S. (2022). Reiki Therapy for Very Young Hospitalized Children Receiving Palliative Care. *Journal of Pediatric Hematology/Oncology Nursing, 39*(1), 15-29. doi:http://dx.doi.org/10.1177/27527530211059435

Tiwari, A. N., Bansal, M., Manju, V. M., Joshi, P., Sinha, A., Hari, P., & Bagga, A. (2015). A Comparative Study to Find out the Health Related Quality of Life of Children with End Stage Renal Disease on Various Renal Replacement therapies: Self and Parental Perception. *International Journal of Nursing Education, 7*(2), 142-146. doi:10.5958/0974-9357.2015.00092.6

Tomlinson, D., Dupuis, L. L., Gibson, P., Johnston, D. L., Portwine, C., Baggott, C., . . . Sung, L. (2014). Initial development of the Symptom Screening in Pediatrics Tool (SSPedi). *Supportive Care in Cancer, 22*(1), 71-75. doi:http://dx.doi.org/10.1007/s00520-013-1945-x

Tomlinson, D., Hendershot, E., Bartels, U., Maloney, A. M., Armstrong, C., Wrathall, G., & Sung, L. (2011). Concordance between couples reporting their child's quality of life and their decision making in pediatric oncology palliative care. *Journal of Pediatric Oncology Nursing, 28*(6), 319-325. doi:https://dx.doi.org/10.1177/1043454211418666

Tomlinson, D., Hinds, P. S., Bartels, U., Hendershot, E., & Sung, L. (2011). Parent reports of quality of life for pediatric patients with cancer with no realistic chance of cure. *Journal of Clinical Oncology, 29*(6), 639-645. doi:https://dx.doi.org/10.1200/JCO.2010.31.4047

Tomlinson, D., Schechter, T., Mairs, M., Loves, R., Herman, D., Hopkins, E., . . . Sung, L. (2021). Finalising the administration of co-SSPedi, a dyad approach to symptom screening for paediatric patients receiving cancer treatments. *BMJ supportive & palliative care, 23*, 23. doi:https://dx.doi.org/10.1136/bmjspcare-2021-003169

Ullrich, C. K., Dussel, V., Hilden, J. M., Sheaffer, J. W., Moore, C. L., Berde, C. B., & Wolfe, J. (2010). Fatigue in children with cancer at the end of life. *Journal of pain and symptom management, 40*(4), 483-494. doi:https://dx.doi.org/10.1016/j.jpainsymman.2010.02.020

Ullrich, C. K., Dussel, V., Orellana, L., Kang, T. I., Rosenberg, A. R., Feudtner, C., & Wolfe, J. (2018). Self-reported fatigue in children with advanced cancer: Results of the PediQUEST study. *Cancer, 124*(18), 3776-3783. doi:https://dx.doi.org/10.1002/cncr.31639

Unay, M., Onder, A., Gizli Coban, O., Atalay, A., Surer Adanir, A., Artan, R., & Ozatalay, E. (2020). Psychopathology, quality of life, and related factors in pediatric liver transplantation candidates and recipients. *Pediatric Transplantation, 24*(1). doi:http://dx.doi.org/10.1111/petr.13633

Vahsen, N., Broder, A., Hraska, V., & Schneider, M. (2018). Neurodevelopmental Outcome in Children With Single Ventricle After Total Cavopulmonary Connection. *Klinische Padiatrie, 230*(1), 24-30. doi:https://dx.doi.org/10.1055/s-0043-120526

Van Cleve, L., Muñoz, C. E., Riggs, M. L., Bava, L., & Savedra, M. (2012). Pain Experience in Children With Advanced Cancer. *Journal of Pediatric Oncology Nursing, 29*(1), 28-36. doi:10.1177/1043454211432295

Van Cleve, L., Muñoz, C. E., Savedra, M., Riggs, M., Bossert, E., Grant, M., & Adlard, K. (2012). Symptoms in children with advanced cancer: child and nurse reports. *Cancer Nursing, 35*(2), 115-125. doi:10.1097/ncc.0b013e31821aedba

Varni, J. W., Limbers, C. A., & Burwinkle, T. M. (2007). Impaired health-related quality of life in children and adolescents with chronic conditions: A comparative analysis of 10 disease clusters and 33 disease categories/severities utilizing the PedsQLTM 4.0 Generic Core Scales. *Health and Quality of Life Outcomes, 5 (no pagination)*. doi:http://dx.doi.org/10.1186/1477-7525-5-43

Vollenbroich, R., Borasio, G. D., Duroux, A., Grasser, M., Brandstatter, M., & Fuhrer, M. (2016). Listening to parents: The role of symptom perception in pediatric palliative home care. *Palliative & supportive care, 14*(1), 13-19. doi:https://dx.doi.org/10.1017/S1478951515000462

Vrijmoet-Wiersma, C. M., Kolk, A. M., Grootenhuis, M. A., Spek, E. M., van Klink, J. M., Egeler, R. M., . . . Koopman, H. M. (2009). Child and parental adaptation to pediatric stem cell transplantation. *Supportive Care in Cancer, 17*(6), 707-714. doi:https://dx.doi.org/10.1007/s00520-008-0544-8

Weaver, M., Wichman, C., Darnall, C., Bace, S., Vail, C., & MacFadyen, A. (2018). Proxy-Reported Quality of Life and Family Impact for Children Followed Longitudinally by a Pediatric Palliative Care Team. *Journal of palliative medicine, 21*(2), 241-244. doi:https://dx.doi.org/10.1089/jpm.2017.0092

Weaver, M. S., Darnall, C., Bace, S., Vail, C., MacFadyen, A., & Wichman, C. (2017). Trending Longitudinal Agreement between Parent and Child Perceptions of Quality of Life for Pediatric Palliative Care Patients. *Children, 4*(8), 01. doi:https://dx.doi.org/10.3390/children4080065

Weaver, M. S., Hanna, R., Hetzel, S., Patterson, K., Yuroff, A., Sund, S., . . . Halanski, M. A. (2020). A Prospective, Crossover Survey Study of Child- and Proxy-Reported Quality of Life According to Spinal Muscular Atrophy Type and Medical Interventions. *Journal of Child Neurology, 35*(5), 322-330. doi:https://dx.doi.org/10.1177/0883073819900463

Weaver, M. S., Robinson, J., & Wichman, C. (2020). Aromatherapy improves nausea, pain, and mood for patients receiving pediatric palliative care symptom-based consults: A pilot design trial. *Palliative & supportive care, 18*(2), 158-163. doi:https://dx.doi.org/10.1017/S1478951519000555

Weaver, M. S., Shostrom, V. K., Neumann, M. L., Robinson, J. E., & Hinds, P. S. (2021). Homestead together: Pediatric palliative care telehealth support for rural children with cancer during home-based end-of-life care. *Pediatric blood & cancer, 68*(4), e28921. doi:https://dx.doi.org/10.1002/pbc.28921

Weaver, M. S., Wang, J., Greenzang, K. A., McFatrich, M., & Hinds, P. S. (2022). The predictive trifecta? Fatigue, pain, and anxiety severity forecast the suffering profile of children with cancer. *Supportive Care in Cancer, 30*(3), 2081-2089. doi:https://dx.doi.org/10.1007/s00520-021-06622-x

Weekly, T., Riley, B., Wichman, C., Tibbits, M., & Weaver, M. (2019). Impact of a Massage Therapy Intervention for Pediatric Palliative Care Patients and Their Family Caregivers. *Journal of palliative care, 34*(3), 164-167. doi:https://dx.doi.org/10.1177/0825859718810727

Wiener, L., Battles, H., Zadeh, S., Widemann, B. C., & Pao, M. (2017). Validity, specificity, feasibility and acceptability of a brief pediatric distress thermometer in outpatient clinics. *Psycho-Oncology, 26*(4), 461-468. doi:https://dx.doi.org/10.1002/pon.4038

Williams, P. D., Schmideskamp, J., Ridder, E. L., & Williams, A. R. (2006). Symptom monitoring and dependent care during cancer treatment in children: Pilot study. *Cancer Nursing, 29*(3), 188-197. doi:http://dx.doi.org/10.1097/00002820-200605000-00004

Wolfe, J., Orellana, L., Cook, E. F., Ullrich, C., Kang, T., Geyer, J. R., . . . Dussel, V. (2014). Improving the care of children with advanced cancer by using an electronic patient-reported feedback intervention: results from the PediQUEST randomized controlled trial. *Journal of Clinical Oncology, 32*(11), 1119-1126. doi:https://dx.doi.org/10.1200/JCO.2013.51.5981

Wolfe, J., Orellana, L., Ullrich, C., Cook, E. F., Kang, T. I., Rosenberg, A., . . . Dussel, V. (2015). Symptoms and distress in children with advanced cancer: Prospective patient-reported outcomes from the PediQUEST study. *Journal of Clinical Oncology, 33*(17), 1928-1935. doi:http://dx.doi.org/10.1200/JCO.2014.59.1222
