# Supplementary material for: Patient-reported outcome measures in children, adolescents, and young adults with palliative care needs—a scoping review
Source: BMC Palliat Care. 2023 Oct 6;22:148. doi: 10.1186/s12904-023-01271-9 (PMC10557323; doi:10.1186/s12904-023-01271-9)
Supplement: Supplementary file 5 — Additional file 5. A complete list of identified PROMs. [file 12904_2023_1271_MOESM5_ESM.docx]

| Name and abbreviation of PROM | Phenomena captured | Used by (N) | Reference |
| --- | --- | --- | --- |
| Baxter Retching Faces (BARF) | Nausea | 2 | (1, 2) |
| Benefit and Burden Scale for Children | Benefit and burden | 1 | (3) |
| Benefit finding scale for children (BFSC) | Benefit | 1 | (4) |
| Body outline | Pain location | 1 | (5) |
| Brief pain inventory (BPI) | Pain | 1 | (6) |
| Brief symptom inventory 18 (BSI-18) | Psychological symptoms | 1 | (7) |
| Child health status one item | Health status | 1 | (8) |
| Childhood Fatigue Scales | Fatigue | 1 | (7) |
| Childhood health assessment questionnaire pain (CHAQ pain) | Pain | 1 | (9) |
| Children’s Anxiety and Pain Scale (CAPS) | Anxiety and pain intensity | 1 | (1) |
| Children’s Comfort Daisies | Comfort | 1 | (10) |
| Children’s Comfort Line Visual Analog Scale | Comfort | 1 | (10) |
| Children’s Comfort 1-item | Comfort | 1 | (10) |
| Children’s Depression Inventory (CDI) | Depressive symptoms | 4 | (4, 7, 11, 12) |
| Children’s Health Questionnaire | HRQL | 1 | (4) |
| Children's palliative outcome scale (CPOS) | Palliative outcomes | 2 | (13, 14) |
| COMFORT-B Scale | Pain and distress | 1 | (15) |
| COPD assessment Test (CAT) | Respiratory-specific QOL | 1 | (16) |
| Connor Davidson Resilience Scale (CDRISC-10) | Resilience | 1 | (3) |
| Dalhousie Dyspnea Scale | Dyspnea | 1 | (2) |
| Distress thermometer | Distress | 3 | (2, 7, 17) |
| ePROtect | Symptoms | 1 | (18) |
| FLACC HCP | Pain | 2 | (2, 19) |
| FACES pictorial scale to assess pain | Pain | 1 | (2) |
| Generic Children's QoL Measure (GCQ) | QOL | 1 | (20) |
| General Health Assessment for Children (GHAC) | QOL | 1 | (21) |
| Hospital Anxiety and Depression Scale (HADS) | Anxiety and depressive symptoms | 1 | (22) |
| Inventory for the assessment of the QOL in children and adolescents (ILK). | QOL | 1 | (23) |
| Kessler 6 Psychological Distress Scale (K-6) | Distress | 1 | (3) |
| Kidscreen-52 | HRQL | 2 | (24, 25) |
| McMurtry Faces Anxiety Scale | Anxiety symptoms | 1 | (2) |
| Memorial Symptom Assessment Scale (MSAS) | Symptoms | 3 | (5, 26, 27) |
| Nurse-documented Neonatal Pain, Agitation and Sedation Scale (N-PASS) | Pain, agitation, and sedation | 2 | (15, 19) |
| Nurse Perceptions of Infant Well-Being Survey | Well-being (Suffering, symptoms and QOL) | 1 | (28) |
| Pediatric Advanced Care-Quality of Life Scale (PAC-QoL) | QOL | 1 | (29) |
| Pediatric Quality of Life Inventory^TM^ (PedsQL) core (various versions) | HRQL | 32 | (3, 6, 8, 12, 16, 17, 30-55) |
| PedsQL 3.0 Brain Tumor Scale | Brain tumor related HRQL | 1 | (39) |
| PedsQL Cancer Module | Cancer-related HRQL | 9 | (3, 11, 47, 49, 51, 52, 56-58) |
| PedsQL End-Stage Renal Disease (ESRD) (various versions) | ESRD-related HRQL | 12 | (20, 30, 32, 33, 41, 42, 59-64) |
| PedsQL multidimensional fatigue scale | Fatigue | 6 | (9, 11, 47, 49, 51, 52) |
| PedsQL 3.0 Neuromuscular Module | Neuromuscular-related HRQL | 1 | (65) |
| Quality of life for primary ciliary dyskinesia (QOL-PCD) | PCD-related HRQL | 1 | (16) |
| Pediatric Patient-Reported Outcomes version of the Common Terminology Criteria for Adverse Events (Ped-PRO-CTCAE) | Adverse events | 3 | (66-68) |
| PediQuest-Memorial Symptom Assessment Scale (PQ-MSAS) | Symptoms | 7 | (37, 45, 69-73) |
| PediQuest (PQ) with PedsQL 4.0 and PQ-MSAS | HRQL and symptoms | 2 | (74, 75) |
| Posttraumatic Stress Disorder Reaction Index (PTSDI) | Posttraumatic Stress Disorder Reaction | 1 | (4) |
| Pre-school Pediatric Cardiac Quality of Life Inventory | Cardiac-related QOL | 1 | (76) |
| Patient Reported Outcomes Measurement Information System (PROMIS) pain intensity | Pain intensity | 1 | (77) |
| PROMIS pain interference | Pain interference | 4 | (6, 68, 77, 78) |
| PROMIS pediatric anger | Co-occurring symptoms/ Anger | 1 | (77) |
| PROMIS anxiety | Co-occurring symptoms/ Anxiety | 3 | (68, 77, 78) |
| PROMIS Depressive symptoms | Co-occurring symptoms/ depressive symptoms | 3 | (68, 77, 78) |
| PROMIS fatigue | Co-occurring symptoms/ fatigue | 3 | (68, 77, 78) |
| PROMIS mobility | Mobility | 1 | (68) |
| PROMIS profile (mobility, fatigue, pain interference, peer relationships, anxiety, and depressive symptoms) | Multidimensional | 3 | (79-81) |
| PROMIS health assessment questionnaire (not specified or referred to domains) | Pain | 1 | (9) |
| PROMIS Pediatric Psychological Stress | Psychological stress | 1 | (66) |
| SCARED screen for anxiety related emotional disorders | Anxiety symptoms | 1 | (12) |
| Scheduled evaluation of individual quality of life (SEIQoL) | QOL | 1 | (13) |
| Short-Form-36 (SF-36) medical outcome study | QOL | 1 | (82) |
| Sinus and Nasal Quality of Life Survey (SN-5) | Nasal-related QOL | 1 | (16) |
| Snyder hope scale | Hope | 1 | (22) |
| Study specific instrument based on the Memorial Symptom Assessment Scale to measure perceptions of infant symptoms and suffering | Infant symptoms and suffering | 1 | (83) |
| Study specific items assessing pain characteristics and pain management | Pain characteristics and pain management | 1 | (77) |
| Study specific measure with 183 items on parental perspectives on multiple domains 9regarding the care of children, adolescents, and young adults with complex chronic conditions | Child suffering | 1 | (84) |
| Study specific, items on palliative course, side effects of radiotherapy | Side effects | 1 | (85) |
| Study specific assessing symptom burden and suffering | Symptom burden and suffering | 1 | (86) |
| Study specific symptom assessment questions based on ESAS, MSAS and PQ-MSAS | Symptoms | 1 | (87) |
| Study specific, 3 primary symptoms questions to identify primary symptoms at last days/ death | Symptoms end-of-life | 1 | (88) |
| Study specific overall health. | Overall health | 1 | (46) |
| Study specific perceived life threat | Life threat | 1 | (11) |
| Study specific on symptoms and suffering, wide range, respiratory distress, pain, agitation, lethargy | Symptoms and suffering | 1 | (19) |
| Study specific 390-item semi-structured questionnaire on symptoms such | Symptoms | 1 | (89) |
| Study specific on symptom perception and treatment | Symptom perception and treatment | 1 | (90) |
| Symptom Screening in Pediatrics Tool (SSPedi) | Symptoms | 4 | (35, 91-93) |
| Therapy-Related Symptom Checklists (TRSC) | Symptoms related to therapy | 1 | (94) |
| “To lose a child” | Well-being last month of life | 1 | (95) |
| TNO-AZL Questionnaires for Children's Health-Related Quality of Life (TACQOL) | HRQL | 2 | (96, 97) |
| Vécu et Santé Perçue de l’Adolescent et l’Enfant (VSP-A) | QOL | 1 | (98) |
| Wong-Baker (FACES) | Pain | 2 | (1, 7) |

**References**

1. Weaver MS, Robinson J, Wichman C. Aromatherapy improves nausea, pain, and mood for patients receiving pediatric palliative care symptom-based consults: A pilot design trial. Palliative & Supportive Care. 2020;18(2):158-63.

2. Weekly T, Riley B, Wichman C, Tibbits M, Weaver M. Impact of a Massage Therapy Intervention for Pediatric Palliative Care Patients and Their Family Caregivers. Journal of Palliative Care. 2019;34(3):164-7.

3. Lau N, Bradford MC, Steineck A, Scott S, Bona K, Yi-Frazier JP, et al. Examining key sociodemographic characteristics of adolescents and young adults with cancer: A post hoc analysis of the Promoting Resilience in Stress Management randomized clinical trial. Palliative Medicine. 2020;34(3):336-48.

4. Phipps S, Peasant C, Barrera M, Alderfer MA, Huang Q, Vannatta K. Resilience in children undergoing stem cell transplantation: results of a complementary intervention trial. Pediatrics. 2012;129(3):e762-70.

5. Van Cleve L, Muñoz CE, Riggs ML, Bava L, Savedra M. Pain Experience in Children With Advanced Cancer. Journal of Pediatric Oncology Nursing. 2012;29(1):28-36.

6. Jibb LA, Stevens BJ, Nathan PC, Seto E, Cafazzo JA, Johnston DL, et al. Implementation and preliminary effectiveness of a real-time pain management smartphone app for adolescents with cancer: A multicenter pilot clinical study. Pediatric Blood & Cancer. 2017;64(10).

7. Wiener L, Battles H, Zadeh S, Widemann BC, Pao M. Validity, specificity, feasibility and acceptability of a brief pediatric distress thermometer in outpatient clinics. Psycho-Oncology. 2017;26(4):461-8.

8. Thrane SE, Williams E, Grossoehme DH, Friebert S. Reiki Therapy for Very Young Hospitalized Children Receiving Palliative Care. Journal of Pediatric Hematology/Oncology Nursing. 2022;39(1):15-29.

9. Haller C, Song W, Cimms T, Chen CY, Whitley CB, Wang RY, et al. Individual heat map assessments demonstrate vestronidase alfa treatment response in a highly heterogeneous mucopolysaccharidosis VII study population. Jimd Reports. 2019;49(1):53-62.

10. Lafond DA, Kelly KP, Hinds PS, Sill A, Michael M. Establishing Feasibility of Early Palliative Care Consultation in Pediatric Hematopoietic Stem Cell Transplantation. Journal of Pediatric Oncology Nursing. 2015;32(5):265-77.

11. Schwartz LA, Brumley LD. What a Pain: The Impact of Physical Symptoms and Health Management on Pursuit of Personal Goals Among Adolescents with Cancer. Journal of Adolescent & Young Adult Oncology. 2017;6(1):142-9.

12. Unay M, Onder A, Gizli Coban O, Atalay A, Surer Adanir A, Artan R, et al. Psychopathology, quality of life, and related factors in pediatric liver transplantation candidates and recipients. Pediatric Transplantation. 2020;24(1).

13. Friedel M, Brichard B, Boonen S, Tonon C, De Terwangne B, Bellis D, et al. Face and Content Validity, Acceptability, and Feasibility of the Adapted Version of the Children's Palliative Outcome Scale: A Qualitative Pilot Study. Journal of Palliative Medicine. 2020;15:15.

14. Namisango E, Bristowe K, Murtagh FE, Downing J, Powell RA, Atieno M, et al. Face and content validity, acceptability, feasibility, and implementability of a novel outcome measure for children with life-limiting or life-threatening illness in three sub-Saharan African countries. Palliative Medicine. 2022:2692163221099583.

15. Fortney CA, Sealschott SD, Pickler RH. Behavioral Observation of Infants With Life-Threatening or Life-Limiting Illness in the Neonatal Intensive Care Unit. Nursing Research. 2020;69(5S Suppl 1):S29-S35.

16. Behan L, Leigh MW, Dell SD, Quittner AL, Hogg C, Lucas JS. Validation of pediatric health-related quality of life instruments for primary ciliary dyskinesia (QOL-PCD). Pediatric Pulmonology. 2019;54(12):2011-20.

17. Schulte F, Russell KB, Pelletier W, Scott-Lane L, Guilcher GMT, Strother D, et al. Screening for psychosocial distress in pediatric cancer patients: An examination of feasibility in a single institution. Pediatric Hematology and Oncology. 2019;36(3):125-37.

18. Meryk A, Kropshofer G, Hetzer B, Riedl D, Lehmann J, Rumpold G, et al. Implementation of daily patient-reported outcome measurements to support children with cancer. Pediatric Blood & Cancer. 2021;68(11):e29279.

19. Shultz EL, Switala M, Winning AM, Keim MC, Baughcum AE, Gerhardt CA, et al. Multiple Perspectives of Symptoms and Suffering at End of Life in the NICU. Advances in Neonatal Care. 2017;17(3):175-83.

20. Heath J, Norman P, Christian M, Watson A. Measurement of quality of life and attitudes towards illness in children and young people with chronic kidney disease. Quality of Life Research. 2017;26(9):2409-19.

21. Lyon ME, Williams PL, Woods ER, Hutton N, Butler AM, Sibinga E, et al. Do-not-resuscitate orders and/or hospice care, psychological health, and quality of life among children/adolescents with acquired immune deficiency syndrome. Journal of Palliative Medicine. 2008;11(3):459-69.

22. Fladeboe KM, O'Donnell MB, Barton KS, Bradford MC, Steineck A, Junkins CC, et al. A novel combined resilience and advance care planning intervention for adolescents and young adults with advanced cancer: A feasibility and acceptability cohort study. Cancer. 2021;127(23):4504-11.

23. Vahsen N, Broder A, Hraska V, Schneider M. Neurodevelopmental Outcome in Children With Single Ventricle After Total Cavopulmonary Connection. Klinische Padiatrie. 2018;230(1):24-30.

24. Dotis J, Pavlaki A, Printza N, Stabouli S, Antoniou S, Gkogka C, et al. Quality of life in children with chronic kidney disease. Pediatric Nephrology. 2016;31(12):2309-16.

25. Houwen-van Opstal SL, Jansen M, van Alfen N, de Groot IJ. Health-related quality of life and its relation to disease severity in boys with Duchenne muscular dystrophy: satisfied boys, worrying parents--a case-control study. Journal of Child Neurology. 2014;29(11):1486-95.

26. Van Cleve L, Muñoz CE, Savedra M, Riggs M, Bossert E, Grant M, et al. Symptoms in children with advanced cancer: child and nurse reports. Cancer Nursing. 2012;35(2):115-25.

27. Weaver MS, Shostrom VK, Neumann ML, Robinson JE, Hinds PS. Homestead together: Pediatric palliative care telehealth support for rural children with cancer during home-based end-of-life care. Pediatric Blood & Cancer. 2021;68(4):e28921.

28. Fortney CA, Pratt M, Dunnells ZDO, Rausch JR, Clark OE, Baughcum AE, et al. Perceived Infant Well-Being and Self-Reported Distress in Neonatal Nurses. Nursing Research. 2020;69(2):127-32.

29. Morley TE, Cataudella D, Fernandez CV, Sung L, Johnston DL, Nesin A, et al. Development of the Pediatric Advanced Care Quality of Life Scale (PAC-QoL): evaluating comprehension of items and response options. Pediatric Blood & Cancer. 2014;61(10):1835-9.

30. De Bruyne E, Eloot S, Vande Walle J, Raes A, Van Biesen W, Goubert L, et al. Validity and reliability of the Dutch version of the PedsQL TM 3.0 End Stage Renal Disease Module in children with chronic kidney disease in Belgium. Pediatric Nephrology. 2022;37(5):1087-96.

31. Derridj N, Bonnet D, Calderon J, Amedro P, Bertille N, Lelong N, et al. Quality of Life of Children Born with a Congenital Heart Defect. Journal of Pediatrics. 2022;244:148-53.e5.

32. El Shafei AM, Soliman Hegazy I, Fadel FI, Nagy EM. Assessment of Quality of Life among Children with End-Stage Renal Disease: A Cross-Sectional Study. Journal of Environmental and Public Health. 2018;2018:8565498.

33. Goldstein SL, Graham N, Warady BA, Seikaly M, McDonald R, Burwinkle TM, et al. Measuring health-related quality of life in children with ESRD: performance of the generic and ESRD-specific instrument of the Pediatric Quality of Life Inventory (PedsQL). American Journal of Kidney Diseases. 2008;51(2):285-97.

34. Hays RM, Valentine J, Haynes G, Geyer JR, Villareale N, McKinstry B, et al. The Seattle pediatric palliative care project: Effects on family satisfaction and health-related quality of life. Journal of Palliative Medicine. 2006;9(3):716-28.

35. Hoffmann S, Schraut R, Kroll T, Scholz W, Belova T, Erhardt J, et al. AquaScouts: ePROs Implemented as a Serious Game for Children With Cancer to Support Palliative Care. Frontiers in Digital Health. 2021;3:730948.

36. Huang I-C, Wen P-S, Revicki DA, Shenkman EA. Quality of life measurement for children with life-threatening conditions: limitations and a new framework. Child indicators research. 2011;4(1):145-60.

37. Ilowite MF, Al-Sayegh H, Ma C, Dussel V, Rosenberg AR, Feudtner C, et al. The relationship between household income and patient-reported symptom distress and quality of life in children with advanced cancer: A report from the PediQUEST study. Cancer. 2018;124(19):3934-41.

38. Limbers CA, Neighbors K, Martz K, Bucuvalas JC, Webb T, Varni JW, et al. Health-related quality of life in pediatric liver transplant recipients compared with other chronic disease groups. Pediatric Transplantation. 2011;15(3):245-53.

39. Mandrell BN, Baker J, Levine D, Gattuso J, West N, Sykes A, et al. Children with minimal chance for cure: parent proxy of the child's health-related quality of life and the effect on parental physical and mental health during treatment. Journal of Neuro-Oncology. 2016;129(2):373-81.

40. Mellion K, Uzark K, Cassedy A, Drotar D, Wernovsky G, Newburger JW, et al. Health-related quality of life outcomes in children and adolescents with congenital heart disease. Journal of Pediatrics. 2014;164(4):781-8.e1.

41. Neul SK, Minard CG, Currier H, Goldstein SL. Health-related quality of life functioning over a 2-year period in children with end-stage renal disease. Pediatric Nephrology. 2013;28(2):285-93.

42. Park KS, Cho MH, Ha IS, Kang HG, Cheong HI, Park YS, et al. Validity and reliability of the Korean version of the pediatric quality of life ESRD module. Health & Quality of Life Outcomes. 2012;10:59.

43. Parsons SK, Fairclough DL, Wang J, Hinds PS. Comparing longitudinal assessments of quality of life by patient and parent in newly diagnosed children with cancer: the value of both raters' perspectives. Quality of Life Research. 2012;21(5):915-23.

44. Rensen N, Steur LMH, Schepers SA, Merks JHM, Moll AC, Kaspers GJL, et al. Determinants of health-related quality of life proxy rating disagreement between caregivers of children with cancer. Quality of Life Research. 2020;29(4):901-12.

45. Requena ML, Avery M, Feraco AM, Uzal LG, Wolfe J, Dussel V. Normalization of Symptoms in Advanced Child Cancer: The PediQUEST-Response Case Study. Journal of Pain and Symptom Management. 2022;63(4):548-62.

46. Roizen M, Rodriguez S, Bauer G, Medin G, Bevilacqua S, Varni JW, et al. Initial validation of the Argentinean Spanish version of the PedsQL 4.0 Generic Core Scales in children and adolescents with chronic diseases: acceptability and comprehensibility in low-income settings. Health & Quality of Life Outcomes. 2008;6:59.

47. Salaverria C, Plenert E, Vasquez R, Fuentes-Alabi S, Tomlinson GA, Sung L. Paediatric relapsed acute leukaemia: curative intent chemotherapy improves quality of life. BMJ supportive & palliative care. 2021;17:17.

48. Splinter A, Tjaden LA, Haverman L, Adams B, Collard L, Cransberg K, et al. Children on dialysis as well as renal transplanted children report severely impaired health-related quality of life. Quality of Life Research. 2018;27(6):1445-54.

49. Stenmarker E, Mellgren K, Matus M, Schroder Hakansson A, Stenmarker M. Health-related quality of life, culture and communication: a comparative study in children with cancer in Argentina and Sweden. Journal of Patientreported Outcomes. 2018;2(1):49.

50. Tanasansuttiporn J, Oofuvong M, Wasinwong W, Chittithavorn V, Duangpakdee P, Jarutach J, et al. Predictors of Health-Related Quality of Life in Children with Cyanotic Heart Disease Who Underwent Palliative and Total Repair. Congenital Heart Disease. 2022;17(3):245-67.

51. Tomlinson D, Hendershot E, Bartels U, Maloney AM, Armstrong C, Wrathall G, et al. Concordance between couples reporting their child's quality of life and their decision making in pediatric oncology palliative care. Journal of Pediatric Oncology Nursing. 2011;28(6):319-25.

52. Tomlinson D, Hinds PS, Bartels U, Hendershot E, Sung L. Parent reports of quality of life for pediatric patients with cancer with no realistic chance of cure. Journal of Clinical Oncology. 2011;29(6):639-45.

53. Varni JW, Limbers CA, Burwinkle TM. Impaired health-related quality of life in children and adolescents with chronic conditions: A comparative analysis of 10 disease clusters and 33 disease categories/severities utilizing the PedsQLTM 4.0 Generic Core Scales. Health and Quality of Life Outcomes. 2007;5 (no pagination).

54. Weaver M, Wichman C, Darnall C, Bace S, Vail C, MacFadyen A. Proxy-Reported Quality of Life and Family Impact for Children Followed Longitudinally by a Pediatric Palliative Care Team. Journal of Palliative Medicine. 2018;21(2):241-4.

55. Weaver MS, Darnall C, Bace S, Vail C, MacFadyen A, Wichman C. Trending Longitudinal Agreement between Parent and Child Perceptions of Quality of Life for Pediatric Palliative Care Patients. Children. 2017;4(8):01.

56. Akard TF, Dietrich MS, Friedman DL, Wray S, Gerhardt CA, Hendricks-Ferguson V, et al. Randomized Clinical Trial of a Legacy Intervention for Quality of Life in Children with Advanced Cancer. Journal of Palliative Medicine. 2020;30:30.

57. Andriastuti M, Halim PG, Kusrini E, Bangun M. Correlation of Pediatric Palliative Screening Scale and Quality of Life in Pediatric Cancer Patients. Indian Journal of Palliative Care. 2020;26(3):338-41.

58. Rosenberg AR, Bradford MC, Bona K, Shaffer ML, Wolfe J, Baker KS, et al. Hope, distress, and later quality of life among adolescent and young adults with cancer. Journal of Psychosocial Oncology. 2018;36(2):137-44.

59. Alhusaini OA, Wayyani LA, Dafterdar HE, Gamlo MM, Alkhayat ZA, Alghamdi AS, et al. Comparison of quality of life in children undergoing peritoneal dialysis versus hemodialysis. Saudi Medical Journal. 2019;40(8):840-3.

60. Baek HS, Park KS, Ha IS, Kang HG, Cheong HI, Park YS, et al. Impact of end-stage renal disease in children on their parents. Nephrology. 2018;23(8):764-70.

61. Goldstein SL, Rosburg NM, Warady BA, Seikaly M, McDonald R, Limbers C, et al. Pediatric end stage renal disease health-related quality of life differs by modality: a PedsQL ESRD analysis. Pediatric Nephrology. 2009;24(8):1553-60.

62. Obiagwu PN, Sangweni B, Moonsamy G, Khumalo T, Levy C. Health-related quality of life in children and adolescents with end-stage renal disease receiving dialysis in Johannesburg. SAJCH South African Journal of Child Health. 2018;12(2):58-62.

63. Park KS, Hwang YJ, Cho MH, Ko CW, Ha IS, Kang HG, et al. Quality of life in children with end-stage renal disease based on a PedsQL ESRD module. Pediatric Nephrology. 2012;27(12):2293-300.

64. Tiwari AN, Bansal M, Manju VM, Joshi P, Sinha A, Hari P, et al. A Comparative Study to Find out the Health Related Quality of Life of Children with End Stage Renal Disease on Various Renal Replacement therapies: Self and Parental Perception. International Journal of Nursing Education. 2015;7(2):142-6.

65. Weaver MS, Hanna R, Hetzel S, Patterson K, Yuroff A, Sund S, et al. A Prospective, Crossover Survey Study of Child- and Proxy-Reported Quality of Life According to Spinal Muscular Atrophy Type and Medical Interventions. Journal of Child Neurology. 2020;35(5):322-30.

66. Hinds PS, Weaver MS, Withycombe JS, Baker JN, Jacobs SS, Mack JW, et al. Subjective Toxicity Profiles of Children in Treatment for Cancer: A New Guide to Supportive Care? Journal of Pain and Symptom Management. 2020;20:20.

67. Leahy AB, Schwartz LA, Li Y, Reeve BB, Bekelman JE, Aplenc R, et al. Electronic symptom monitoring in pediatric patients hospitalized for chemotherapy. Cancer. 2021;127(16):2980-9.

68. Weaver MS, Wang J, Greenzang KA, McFatrich M, Hinds PS. The predictive trifecta? Fatigue, pain, and anxiety severity forecast the suffering profile of children with cancer. Supportive Care in Cancer. 2022;30(3):2081-9.

69. Boyden JY, Hill DL, Nye RT, Bona K, Johnston EE, Hinds P, et al. Pediatric Palliative Care Parents' Distress, Financial Difficulty, and Child Symptoms. Journal of Pain and Symptom Management. 2022;63(2):271-82.

70. Feudtner C, Nye R, Hill DL, Hall M, Hinds P, Johnston EE, et al. Polysymptomatology in Pediatric Patients Receiving Palliative Care Based on Parent-Reported Data. JAMA Network Open. 2021;4(8):e2119730.

71. Montgomery KE, Raybin JL, Ward J, Balian C, Gilger E, Murray P, et al. Using Patient-Reported Outcomes to Measure Symptoms in Children with Advanced Cancer. Cancer Nursing. 2020;43(4):281-9.

72. Ullrich CK, Dussel V, Orellana L, Kang TI, Rosenberg AR, Feudtner C, et al. Self-reported fatigue in children with advanced cancer: Results of the PediQUEST study. Cancer. 2018;124(18):3776-83.

73. Wolfe J, Orellana L, Ullrich C, Cook EF, Kang TI, Rosenberg A, et al. Symptoms and distress in children with advanced cancer: Prospective patient-reported outcomes from the PediQUEST study. Journal of Clinical Oncology. 2015;33(17):1928-35.

74. Rosenberg A, Orellana L, Ullrich C, Kang T, Geyer J, Feudtner C, et al. Quality of life in children with advanced cancer: A report from the pediQUEST study. Journal of Pain and Symptom Management. 2016;52(2):243-53.

75. Wolfe J, Orellana L, Cook EF, Ullrich C, Kang T, Geyer JR, et al. Improving the care of children with advanced cancer by using an electronic patient-reported feedback intervention: results from the PediQUEST randomized controlled trial. Journal of Clinical Oncology. 2014;32(11):1119-26.

76. Heye KN, Knirsch W, Scheer I, Beck I, Wetterling K, Hahn A, et al. Health-related quality of life in pre-school age children with single-ventricle CHD. Cardiology in the Young. 2019;29(2):162-8.

77. Cheng L, Yuan C, Wang J, Stinson J. Pain Reported by Chinese Children during Cancer Treatment: Prevalence, Intensity, Interference, and Management. Cancer Nursing. 2022;45(2):E345-E54.

78. Grossoehme DH, Friebert S, Baker JN, Tweddle M, Needle J, Chrastek J, et al. Association of Religious and Spiritual Factors With Patient-Reported Outcomes of Anxiety, Depressive Symptoms, Fatigue, and Pain Interference Among Adolescents and Young Adults With Cancer. JAMA Network Open. 2020;3(6):e206696.

79. Dobrozsi S, Yan K, Hoffmann R, Panepinto J. Patient-reported health status during pediatric cancer treatment. Pediatric Blood & Cancer. 2017;64(4):04.

80. Ellis GK, Chapman H, Manda A, Salima A, Itimu S, Banda G, et al. Pediatric lymphoma patients in Malawi present with poor health-related quality of life at diagnosis and improve throughout treatment and follow-up across all Pediatric PROMIS-25 domains. Pediatric Blood and Cancer. 2021;68(10):e29257.

81. Selewski DT, Massengill SF, Troost JP, Wickman L, Messer KL, Herreshoff E, et al. Gaining the Patient Reported Outcomes Measurement Information System (PROMIS) perspective in chronic kidney disease: a Midwest Pediatric Nephrology Consortium study. Pediatric Nephrology. 2014;29(12):2347-56.

82. Husson O, Zebrack BJ, Block R, Embry L, Aguilar C, Hayes-Lattin B, et al. Health-Related Quality of Life in Adolescent and Young Adult Patients With Cancer: A Longitudinal Study. Journal of Clinical Oncology. 2017;35(6):652-9.

83. Baughcum AE, Fortney CA, Winning AM, Dunnells ZDO, Humphrey LM, Gerhardt CA. Healthcare Satisfaction and Unmet Needs Among Bereaved Parents in the NICU. Advances in Neonatal Care. 2020;20(2):118-26.

84. DeCourcey DD, Silverman M, Oladunjoye A, Wolfe J. Advance Care Planning and Parent-Reported End-of-Life Outcomes in Children, Adolescents, and Young Adults With Complex Chronic Conditions. Critical Care Medicine. 2019;47(1):101-8.

85. Lee BK, Boyle PJ, Zaslowe-Dude C, Wolfe J, Marcus KJ. Palliative radiotherapy for pediatric patients: Parental perceptions of indication, intent, and outcomes. Pediatric Blood & Cancer. 2020;67(1):e28003.

86. Levine DR, Mandrell BN, Sykes A, Pritchard M, Gibson D, Symons HJ, et al. Patients' and Parents' Needs, Attitudes, and Perceptions About Early Palliative Care Integration in Pediatric Oncology. JAMA Oncology. 2017;3(9):1214-20.

87. Madden K, Magno Charone M, Mills S, Dibaj S, Williams JL, Liu D, et al. Systematic symptom reporting by pediatric palliative care patients with cancer: a preliminary report. Journal of palliative medicine. 2019;22(8):894-901.

88. Pritchard M, Burghen E, Srivastava DK, Okuma J, Anderson L, Powell B, et al. Cancer-related symptoms most concerning to parents during the last week and last day of their child's life. Pediatrics. 2008;121(5):e1301-e9.

89. Ullrich CK, Dussel V, Hilden JM, Sheaffer JW, Moore CL, Berde CB, et al. Fatigue in children with cancer at the end of life. Journal of Pain and Symptom Management. 2010;40(4):483-94.

90. Vollenbroich R, Borasio GD, Duroux A, Grasser M, Brandstatter M, Fuhrer M. Listening to parents: The role of symptom perception in pediatric palliative home care. Palliative & Supportive Care. 2016;14(1):13-9.

91. Szepetowski S, Saultier P, Andre N, Pauly V, Dupuis LL, Sung L, et al. Symptom Screening in Pediatrics Tool in children and adolescents with high-risk malignancies: a pilot study. BMJ supportive & palliative care. 2021;15:15.

92. Tomlinson D, Dupuis LL, Gibson P, Johnston DL, Portwine C, Baggott C, et al. Initial development of the Symptom Screening in Pediatrics Tool (SSPedi). Supportive Care in Cancer. 2014;22(1):71-5.

93. Tomlinson D, Schechter T, Mairs M, Loves R, Herman D, Hopkins E, et al. Finalising the administration of co-SSPedi, a dyad approach to symptom screening for paediatric patients receiving cancer treatments. BMJ supportive & palliative care. 2021;23:23.

94. Williams PD, Schmideskamp J, Ridder EL, Williams AR. Symptom monitoring and dependent care during cancer treatment in children: Pilot study. Cancer Nursing. 2006;29(3):188-97.

95. Lykke C, Ekholm O, Olsen M, Sjogren P. Paediatric end-of-life care - symptoms and problems: parent assessment. BMJ supportive & palliative care. 2021;11:11.

96. Eijsermans RM, Creemers DG, Helders PJ, Schroder CH. Motor performance, exercise tolerance, and health-related quality of life in children on dialysis. Pediatric Nephrology. 2004;19(11):1262-6.

97. Vrijmoet-Wiersma CM, Kolk AM, Grootenhuis MA, Spek EM, van Klink JM, Egeler RM, et al. Child and parental adaptation to pediatric stem cell transplantation. Supportive Care in Cancer. 2009;17(6):707-14.

98. Clave S, Tsimaratos M, Boucekine M, Ranchin B, Salomon R, Dunand O, et al. Quality of life in adolescents with chronic kidney disease who initiate haemodialysis treatment. BMC Nephrology. 2019;20(1):163.
